# Vaccination against Bacterial Infections: Challenges, Progress, and New Approaches with a Focus on Intracellular Bacteria

**DOI:** 10.3390/vaccines10050751

**Published:** 2022-05-10

**Authors:** Anke Osterloh

**Affiliations:** Department of Infection Immunology, Research Center Borstel, Parkallee 22, 23845 Borstel, Germany; aosterloh@fz-borstel.de; Tel.: +49-45-3718-84822

**Keywords:** extracellular and intracellular bacteria, vaccine, immunity, antigens

## Abstract

Many bacterial infections are major health problems worldwide, and treatment of many of these infectious diseases is becoming increasingly difficult due to the development of antibiotic resistance, which is a major threat. Prophylactic vaccines against these bacterial pathogens are urgently needed. This is also true for bacterial infections that are still neglected, even though they affect a large part of the world’s population, especially under poor hygienic conditions. One example is typhus, a life-threatening disease also known as “war plague” caused by *Rickettsia prowazekii*, which could potentially come back in a war situation such as the one in Ukraine. However, vaccination against bacterial infections is a challenge. In general, bacteria are much more complex organisms than viruses and as such are more difficult targets. Unlike comparatively simple viruses, bacteria possess a variety of antigens whose immunogenic potential is often unknown, and it is unclear which antigen can elicit a protective and long-lasting immune response. Several vaccines against extracellular bacteria have been developed in the past and are still used successfully today, e.g., vaccines against tetanus, pertussis, and diphtheria. However, while induction of antibody production is usually sufficient for protection against extracellular bacteria, vaccination against intracellular bacteria is much more difficult because effective defense against these pathogens requires T cell-mediated responses, particularly the activation of cytotoxic CD8^+^ T cells. These responses are usually not efficiently elicited by immunization with non-living whole cell antigens or subunit vaccines, so that other antigen delivery strategies are required. This review provides an overview of existing antibacterial vaccines and novel approaches to vaccination with a focus on immunization against intracellular bacteria.

## 1. Introduction

### 1.1. Extracellular and Intracellular Bacterial Pathogens

The most common bacterial pathogens in the Western world today include *Listeria*, *Salmonella* (*S.*) *enterica* ssp., *Helicobacter* (*H.*) *pylori*, *Escherichia* (*E.*) *coli*, *Staphylococcus* (*S.*) *aureus*, *Streptococcus* (*S.*) *pneumoniae* (pneumococci), *Neisseria* (*N.*) *meningitidis* (meningococci), and *Klebsiella* (*K.*) *pneumoniae*. Causative agents of other bacterial diseases, often acquired upon hospitalization, are *Acinetobacter* (*A.*) *baumanii*, *Clostridioides* (*C.*) *difficile*, and *Pseudomonas* (*P.*) *aeruginosa*. In earlier times, infections with *Clostridium* (*C.*) *tetani* (tetanus), *Vibrio* (*V.*) *cholerae* (cholera), *Corynebacterium* (*C.*) *diphteriae* (diphtheria), *Bordetella* (*B.*) *pertussis* (whooping cough), and *Salmonella enterica* ssp. *enterica* serovar Typhi (typhoid fever)) were also major health problems before vaccines against these pathogens were developed.

The majority of these bacteria are free-living pathogens that exist in the environment and can replicate in the extracellular space. Upon entry into the body, they are usually eliminated by the uptake by phagocytes such as macrophages (MØ), neutrophils, and dendritic cells (DCs) and degraded in lysosomal compartments within these cells. Nevertheless, these bacteria can be dangerous, e.g., via the release of harmful toxins or by triggering excessive inflammatory reactions that damage cells and tissues. 

In contrast to free-living bacterial pathogens, intracellular bacteria have evolved mechanisms to escape the process of lysosomal degradation within target cells to replicate within these cells. The lifestyle of intracellular bacteria requires different mechanisms of immune defense, in particular the activation of T cells, especially CD8^+^ T cells. The following paragraphs summarize the knowledge about the lifestyle of these pathogens and immune defense mechanisms because knowledge of protective immune responses as well as the lifestyle of these bacteria is an essential prerequisite for vaccine development. 

Facultative intracellular bacteria can exist in the extracellular environment as well as within host cells, while obligate intracellular bacteria strictly depend on host cells for survival and replication. Examples of facultative intracellular bacterial pathogens are *Mycobacterium* (*M.*) *tuberculosis*, *N. meningitidis*, *Legionella* (*L.*) *pneumophila*, *Listeria* (*L.*) *monocytogenes*, *Shigella* (*S.*) *dysenteriae*, *Francisella* (*F.*) *tularensis*, *Bordetella* (*B.*) *pertussis*, and *Bacillus* (*B.*) *anthracis*. In addition, *S. aureus* can infect host cells, although it predominantly appears extracellularly. 

Only a few bacterial pathogens have an obligate intracellular lifestyle and strictly depend on host cells for multiplication. Examples of these obligate intracellular bacteria are *Chlamydia* ssp., *Anaplasma* ssp., *Ehrlichia* ssp., *Coxiella* ssp., and all rickettsial and orientia species. An overview of extracellular, facultative, and obligate intracellular bacterial pathogens and diseases is given in Supplementary Table S1 that also provides information on currently available vaccines (Table S1).

**Figure 1:**
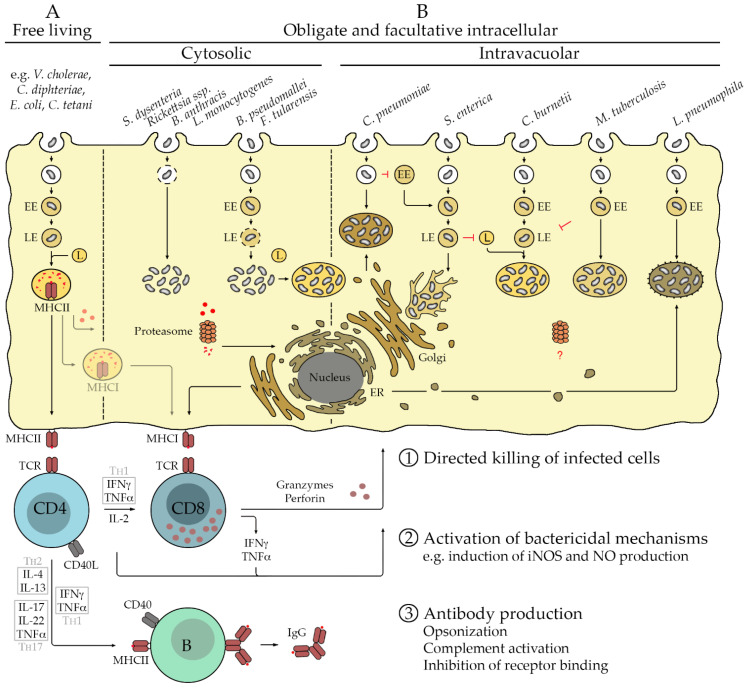
Extra- and intracellular bacteria and immune response. Free-living bacteria are taken up by phagocytes such as MØ and DCs as well as B cells that serve as professional antigen-presenting cells (APCs). Phagosomes develop into early endosomes (EE) and further to late endosomes (LE) that finally fuse with lysosomes (L). The activity of proteases and acidic environments of the Ls results in the degradation of the pathogen and its proteins, fragments of which are bound by MHCII molecules in the lysosomal membrane. MHCII/peptide complexes are presented on the cell surface to CD4^+^ T cells. The protective immune response is dominated by activated CD4^+^ T cells as well as B cells that produce antibodies against surface molecules of the pathogen. Depending on the cytokine environment provided by the APC, CD4^+^ T cells develop to T helper (T_H_) cells, either to T_H_2 cells producing IL-4 and IL-13, T_H_1 cells that secrete IFNγ and TNFα, or T_H_17 cells releasing IL-17, TNFα, and IL-22 that acts on non-immune cells. All T_H_ cells also release IL-2, which promotes T cell proliferation and survival. Activated T_H_ cells interact with activated B cells via the binding of CD40L to CD40 on the B cell surface, initiating the germinal center reaction where immunoglobulin class switch and affinity maturation occurs, so that high-affinity IgG instead of the initial IgM is produced. In addition, memory B cells develop. The cytokines that are produced by different T_H_ cells promote the generation of certain IgG isotypes in this process. Antibodies can act against extracellular bacteria by opsonization for the uptake by phagocytes or direct destruction of the pathogen by complement activation. (**A**). Only a few bacteria replicate exclusively within target cells. These include members of the family of Rickettsiacea, *Chlamydia* (*C.*) *pneumoniae*, and *Coxiella* (*C.*) *burnetii*. Rickettsiae escape from the phagosome via the release of phospholipases that dissolve the phagosomal membrane and replicate free in the cytosol [6]. *C. pneumoniae*, instead, leaves the endocytic route and recruits Golgi-derived vesicles to form a unique compartment for replication that is associated with the microtubule organizing center (MTOC) [7]. Phagosomes containing *C. burnetii*, in turn, fuse with Ls to build a phagolysosomal-like vacuole for bacterial replication. Several other bacteria are facultative intracellular pathogens. Examples are *B. anthracis*, *L*. *monocytogenes*, *B. pseudomallei*, *F. tularensis*, *S. enterica*, *M. tuberculosis*, and *L. pneumophila*. Similar to rickettsiae, *B. anthracis* infects macrophages (MØ) and escapes from the phagosome to replicate free in the cytosol. *L. monocytogenes*, *B. pseudomalleii*, and *F. tularensis* deliberate from LEs and then also replicate free in the cytosol of infected cells. Cytosolic *F. tularensis* may also retranslocate into autolysosome-like vacuoles. In contrast, *S. enterica* inhibits fusion of LEs with Ls and replicates in LE-like compartments that are associated with the MTOC and form filaments [8]. *M. tuberculosis* inhibits maturation of EEs and replicates in LE-like vacuoles, while EEs containing *L. pneumophila* fuse with vesicles derived from the ER to form ribosome-coated compartments for bacterial replication [9]. (**B**). Efficient defense against intracellular pathogens usually requires the activation of cytotoxic CD8^+^ T cells that are capable of the direct killing of infected cells. CD8^+^ T cells are activated by antigenic peptides that derive from cytosolic proteins that are degraded by the proteasome. Peptides are transferred to the ER to be loaded onto MHCI molecules that are presented on the cell surface of all nucleated cells to be recognized by CD8^+^ T cells. Bacterial antigens that are recognized by CD8^+^ T cells may derive predominantly from secreted proteins or surface proteins that are accessible for proteasomal degradation in the cytosol ①. Initial activation of CD8^+^ T cells and defense against intracellular bacteria further require the activation of CD4^+^ T cells, predominantly of the T_H_1 type. These cells support CD8^+^ T cell responses. In addition, CD4^+^ T_H_1 cells (as well as T_H_17 cells and IFNγ-releasing CD8^+^ T cells) induce bactericidal mechanisms such as the induction of nitric oxide synthase (iNOS) and NO production in infected cells via the release of IFNγ and TNFα. In this way, CD4^+^ T_H_1 cells contribute to bacterial elimination ②. Antibodies produced by B cells may play a minor role in the defense against primary infection with intracellular bacteria but can contribute to protection in secondary infection. In addition to the aforementioned mechanisms, antibodies can here participate in defense by the inhibition of the binding of the bacteria to receptors that mediate bacterial uptake into target cells ③. For those bacteria that replicate within cellular compartments and thus are hidden from the cytosol and the proteasome, the activation of CD8^+^ T cell responses during the infection may not be efficient.

Intracellular bacteria use different mechanisms to escape from phagolysosomal degradation to survive and to proliferate within host cells. Depending on the species, the bacteria replicate either free in the cytosol or within certain cellular compartments. Cytosolic replication is observed for *L. monocytogenes*, *S. dysenteriae*, *B. anthracis*, rickettsial species, *Burkholderia* (*B.*) *pseudomallei*, and *F. tularensis*. *L. monocytogenes*, *S. dysenteriae*, *B. anthracis*, and rickettsiae directly escape from the early phagosomal vacuole [1], while *B. pseudomallei* and *F. tularensis* escape from the endosome at a later stage. *B. pseudomallei* liberates from late endosomes after fusion of the phagosome with early endosomes [2], while phagosomes containing *F. tularensis* develop to late endosomes that become acidic before they disrupt to release the bacteria into the cytosol [3]. Apart from cytosolic replication, *F. tularensis* may also retranslocate into vesicles that resemble autolysosomes [2]. Other bacteria replicate in specialized vacuoles. *L. pneumophila* segregates from the endocytic route at the early endosome stage and recruits vesicles from the endoplasmic reticulum (ER) to form ribosome-coated inclusion vacuoles in which the bacteria multiply [2]. *C. pneumonia* avoids fusion of the phagosome with early endosomes and recruits Golgi-derived vesicles to form a compartment for replication [2], while *M. tuberculosis* resides in early endosomes and inhibits fusion with the lysosome and acidification of the vesicle for replication [2]. In contrast, *C. burnetii* multiplies in phagolysome-like acidic vacuoles after fusion of the late endosome with the lysosome [2,4]. Finally, *S. enterica* ssp. *enterica* replicate in late-endosome-like vesicles that recruit preexisting lysosomal proteins but do not fuse with the lysosome, so that the bacteria are excluded from degradation. These *S. enterica*-containing vacuoles migrate and attach to the microtubule-organizing center (MTOC) that nucleates at the Golgi [2,5]. Figure 1 provides an overview of extra- and intracellular bacterial pathogens as well as immune mechanisms that are involved in defense and described in the following section.

### 1.2. Immunological Defense against Extracellular and Intracellular Bacterial Pathogens

Defense against extra- and intracellular bacteria requires different immunological mechanisms. Protection against extracellular bacteria is predominantly mediated by antibodies produced by B cells and CD4^+^ T helper cells that assist B cells to produce high-affinity class-switched IgG instead of IgM. Extracellular bacteria are taken up by phagocytes such as MØ and DCs that also serve as professional antigen-presenting cells (APCs). Upon phagocytosis of the pathogen, phagosomes containing the bacteria mature by fusion with endosomes and finally lysosomes. These provide an acidic environment and several enzymes that are involved in bacterial killing and degradation. In addition, the membrane of the phagolysosomal compartment of APCs contains preformed major histocompatibility complex class II (MHCII) molecules. These are loaded with peptides derived from degraded proteins from the pathogen and transported to the cell surface where CD4^+^ T cells can recognize the bound peptide antigens via the T cell receptor (TCR). Activated APCs that recognize and encounter bacteria upregulate the expression of costimulatory molecules (CD80/CD86) that are required for T cell activation and release cytokines that drive the differentiation of T cells into T_H_1, T_H_2, or T_H_17 cells. The main inducer of IFNγ/TNFα-secreting T_H_1 cells is IL-12. In the absence of this cytokine, either T_H_2 that secretes IL-4 and IL-13 or T_H_17 that releases IL-17 and TNFα, in addition to IL-22, a cytokine that acts on non-immune cells, develop. T_H_17 differentiation further requires the release of IL-23 or the presence of TGFb and IL-6 [10]. Activated CD4^+^ T cells further produce IL-2 that serves as a growth and survival factor for T cells. In this situation, the main function of CD4^+^ T_H_ cells is to interact with activated B cells that have recognized antigen. Binding of CD40L on the surface of activated CD4^+^ T cells with CD40 on the B cell surface induces the germinal center reaction. During this reaction, B cells undergo an immunoglobulin isotype class switch and affinity maturation and start to release high-affinity IgG antibodies instead of IgM, which is initially produced. In addition, memory B cells are generated. The cytokines provided by the T_H_ cells influence the isotype class switch and favor the production of certain antibody isotypes. IL-4 promotes the production of IgG1, while IFNγ promotes the production of IgG2 [11]. Antibodies play a major role in defense against extracellular bacteria and can either induce direct complement-mediated killing of the pathogen or opsonize the bacteria for the uptake by phagocytes to be eliminated (Figure 1).

Defense against intracellular bacteria requires different immunological mechanisms compared to extracellular bacteria, with cytotoxic CD8^+^ T cells playing a central role in addition to T_H_1-biased CD4^+^ T cell responses rather than antibodies. While antigens that are recognized by CD4^+^ T cells usually derive from extracellular material, CD8^+^ T cells recognize peptide antigens that originate from cytosolic proteins that are degraded by the proteasome. These can be misfolded endogenous proteins as well as pathogen-derived proteins, e.g., proteins that are secreted by the pathogen or present on the pathogen surface, which are accessible for the proteasome in the cytosol. Protein fragments resulting from proteasomal degradation are then translocated into the ER via the transporter associated with antigen processing (TAP). In the ER, antigenic peptides are bound to MHCI molecules that are transferred to the cellular surface via the Golgi apparatus to be presented to CD8^+^ T cells (Figure 1). Activated CD8^+^ T cells are capable of the direct killing of infected cells that present specific antigen via MHCI. Killing of target cells by CD8^+^ T cells is mediated by the directed release of granzymes and perforin that induce apoptosis, leading to elimination of the infected cell and the bacteria contained within. In contrast to MHCII, which is exclusively expressed by professional APCs, MHCI molecules are present on all nucleated cells so that CD8^+^ T cells can exert cytotoxic activity against all types of cells. 

The initial activation of functional cytotoxic CD8^+^ T cells further requires CD4^+^ T helper cells that provide IL-2, which is essential for T cell survival and additional cytokines. Usually, CD4^+^ T cells of the T_H_1 type that produce IFNγ and TNFα are required for defense against intracellular bacteria. Apart from assisting in the activation of CD8^+^ T cells (and B cells), CD4^+^ T_H_1 cells themselves can substantially contribute to bacterial killing by activating bactericidal mechanisms in phagocytes and other cells. This is mainly mediated via the induction of inducible nitric oxide synthase (iNOS) via IFNγ and TNFα [12,13,14,15] and subsequent production of bactericidal nitric oxide (NO). B cells generally play a minor role in defense against intracellular bacteria, at least in primary infection. Nonetheless, antibodies produced by B cells may help to protect against secondary infection. Here, in addition to the previously mentioned mechanisms, antibodies can also inhibit receptor-mediated uptake of the pathogen by target cells and in this way contribute to defense.

An efficient vaccine against intracellular bacteria should ideally address the activation of CD8^+^ T cells in addition to CD4^+^ T_H_1 cells and B cells.

### 1.3. Antibiotic-Resistant Bacterial Pathogens and the Urgent Need for New Vaccines

Bacterial infections are usually treated with antibiotics. Many bacteria, however, have developed antibiotic resistance, which is a great threat.

In 2017, the world health organization (WHO) published a list of bacteria for which new antibiotics (or vaccines) are urgently needed. A high priority pathogen is *A. baumanii*, which accounts for approximately 2% of all healthcare-associated infections in the U.S. and Europe [16] and two times higher rates in the Middle East and Asia [17]. On average, around 45% of *A. baumanii* isolates are considered multi-drug resistant. The priority list further names *P. aeruginosa*, enterobacteria, enterococci, *S. aureus*, *H. pylori*, *Campylobacter* ssp., *Salmonellae*, *N. gonorrheae*, *S. pneumoniae*, *H. influenzae*, and *Shigella* ssp. (https://www.who.int/news/item/27-02-2017-who-publishes-list-of-bacteria-for-which-new-antibiotics-are-urgently-needed; accessed on 4 May 2022) (Table S2). Information on estimates of the global incidence of resistant bacterial infections can be found on the WHO page (https://www.who.int/publications/i/item/9789240027336, accessed on 4 May 2022). Table S2 shows specific data from the U.S. that are recorded and published for public access by the Centers for Disease Control and Prevention (CDC) as an example, while clear data from other countries are scarce.

In the U.S., *A. baumanii*, *C. difficile*, Enterobacterales, *N. gonorrhea*, *H. pylori*, Enterococci, *P. aeruginosa*, *Salmonella* spp., *Salmonella* Typhi, *Shigella*, *S. aureus*, *S. pneumoniae*, and *M. tuberculosis* are among the most prevalent bacterial infections with antibiotic resistances. In addition, antibiotic-resistant *Streptococcus* Group A and B are concerning threats, and antibiotic-resistant *M. genitalium* and *B. pertussis* are on the watch list of the CDC (https://www.cdc.gov/drugresistance/biggest-threats.html; accessed on 8 April 2022).

In other countries, especially in the poorer developing countries, numbers of infections with certain bacteria may differ significantly and may be much higher. The same might be true for the occurrence of resistant bacterial pathogens. In addition, little is known about the prevalence of many other bacterial pathogens, although they certainly affect a large number of people worldwide. This is partly because many of these infectious diseases are not reportable, and partly because they are underdiagnosed or (re-) emerging. Examples include rickettsial infections that occur worldwide with increasing incidence and geographic distribution. Infections with *R. typhi* (endemic typhus) and *O. tsutsugamushi* (Scrub typhus) are major causes of severe meningitis and meningoencephalitis with high mortality rates in the Asia–Pacific region [18], and it is estimated that around 1 million people per year acquire infections with *O. tsutsugamushi* [19,20]. Moreover, for some bacterial pathogens, the spectrum of effective antibiotics is limited. In these cases, alternatives are generally rare. An example is again rickettsiae, which responds to only a few antibiotics, with doxycycline being the treatment of choice. The development of resistance to doxycycline is therefore a very significant concern, and there are hints that doxycycline-resistant *O. tsutsugamushi* already occurs [21,22,23]. In addition, treatment of doxycycline-intolerant patients is difficult. 

Furthermore, certain bacteria, including rickettsial species (*R. prowazekii*, *R. rickettsii*) and *B. anthracis*, are classified as potential bioweapons. These bacteria can be genetically manipulated to acquire antibiotic resistance, which is another reason for the urgent need for vaccines against these particular species, in addition to vaccines against several other bacterial infections. 

Most vaccines that have been developed in the past and are in use today target extracellular bacteria. Vaccines against intracellular bacteria are generally rare, and with the exception of a vaccine against Q fever, which is used only in Australia, none are available against obligate intracellular bacteria.

In the following sections, applied and experimental methods of vaccination against bacteria are described, and their applicability to vaccination against intracellular bacterial pathogens is discussed.

## 2. Types of Bacterial Vaccines and the Difficulties of Vaccination against Intracellular Bacterial Pathogens

**Figure 2:**
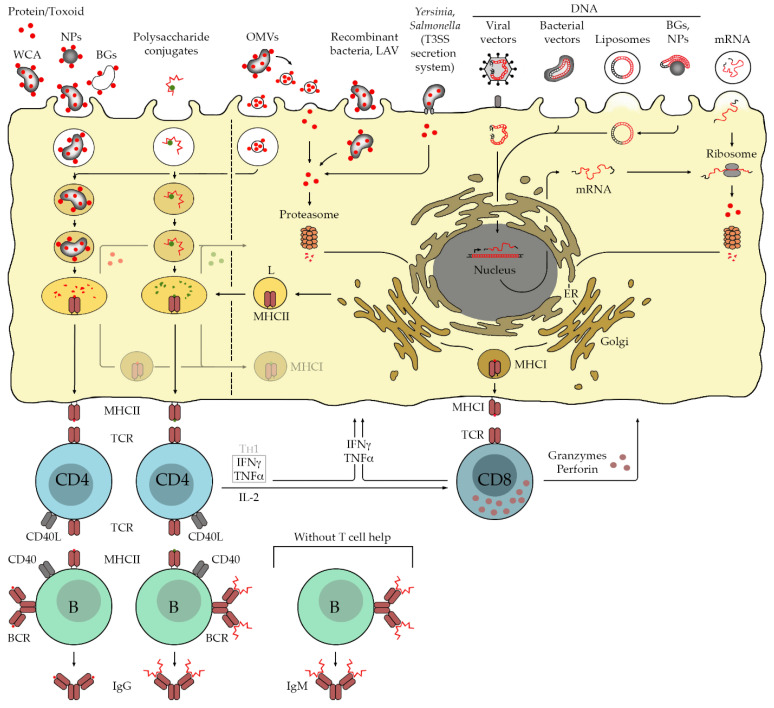
Applied and experimental bacterial vaccines. Today, established bacterial vaccines that are in use are the immunizations with WCA, recombinant proteins including toxoids, polysaccharide/protein conjugates, live attenuated vaccines (LAV), and, since the last few years, also bacterial outer membrane vesicles (OMVs). Vaccination with WCA, bacterial ghosts (BGs), and recombinant proteins/toxoids predominantly results in the processing of protein components for the presentation via MHCII and the activation predominantly of CD4^+^ T cells. In addition, antigen-specific activated B cells produce high-affinity IgG antibodies with the help of CD4^+^ T cells, and a memory response is induced. In the case of polysaccharide/protein conjugates, the carrier protein serves as the protein component that can be recognized by CD4^+^ T cells. This enables B cells to produce high-affinity IgG antibodies against the polysaccharide instead of the production of low-affinity IgM without T cell help. Immunization against intracellular bacterial pathogens requires the activation of cytotoxic CD8^+^ T cells, which is usually not efficiently achieved with these methods. Antigens recognized by CD8^+^ T cells predominantly derive from cytosolic proteins that are degraded by the proteasome and further processed for the presentation via MHCI. A major difficulty of efficient vaccination against intracellular bacterial pathogens lies in the introduction of immunogens into the cytosol of host cells. This can be achieved by the use of OMVs, LAV, viral vectors, bacterial vectors, immunization with DNA or mRNA, and the use of the T3SS translocation system of bacteria such as *Salmonella*. Immunization with OMVs and LAV results in both antigen presentation by MHCII and MHCI molecules for the activation of CD4^+^ and CD8^+^ T cells, whereby the mechanisms of MHCI presentation in the case of OMV immunization are not well understood and may be a result of cross presentation, either by the release of proteins from the lysosome into the cytosol or by fusion of lysosomes with MHCI-containing vesicles. LAV may release proteins into the cytosol. In addition, surface proteins may be accessible to the proteasome for processing via the MHCI presentation pathway. The most frequently used viral vectors for vaccination are adenoviruses and modified vaccinia virus Ankara (MVA). Adenoviruses translocate their double-stranded (ds) DNA genome into the nucleus of non-dividing cells for replication. Viral mRNA transcription products are exported into the cytosol of the infected cells where ribosomal translation occurs. In contrast, MVA, which also carries a dsDNA genome, has a unique replication cycle that is restricted to the cytosol. In both cases, proteins are expressed in the cytosol of infected cells, which has also been shown for bacterial vectors that carry plasmid DNA with eukaryotic expression cassettes for the expression of immunogens. Cytosolic protein expression is also achieved by the direct transfection of target cells with either DNA or mRNA. While mRNA is transferred directly into the cytosol for protein translation, DNA has to enter the nucleus of the target cell for transcription, which is usually more efficiently achieved with viral vectors. Finally, a rather experimental approach to the introduction of antigens into the cytosol of target cells is the use of recombinant attenuated bacteria such as *Salmonella* that possess a T3SS translocation system. This system allows active and direct injection of proteins into the cytosol of target cells. In contrast to the use of WCA and LAV, all other methods generally require knowledge of the immunogenic determinants of the pathogen to prepare recombinant vaccines.

Generally, mainly four types of vaccines against bacterial infections are in use today: inactivated bacterial pathogens (whole cell antigen (WCA)), live attenuated bacterial vaccines (LAV), toxoid and subunit vaccines, and polysaccharide conjugate vaccines. In addition, recent advances in new technologies and experimental approaches of vaccination that may be suitable also for immunization against infections with intracellular bacteria are discussed in the following. These include live recombinant bacteria, the use of outer bacterial membrane vesicles (OMVs), bacterial ghosts (BGs), immunization with nucleotides (DNA, mRNA, viral and bacterial vectors), and nanoparticles (NPs) conjugated with antigens or nucleotides. Table 1 and Figure 2 summarize applied and experimental methods of vaccination against bacterial infections that are described in the following paragraphs in more detail.

### 2.1. Whole Cell Antigen (WCA)

WCA can be obtained by heating, irradiation, or chemical inactivation of the bacteria such as the treatment with formaldehyde or alkylating reagents. In contrast to heat-killing and chemical inactivation, which can potentially mask or destroy antigenic determinants, irradiation might be more promising to obtain intact bacteria and its antigens. WCA immunization predominantly induces the activation of B cells and antibody production as well as the activation of CD4^+^ T cells. Although cross presentation occurs to some extent (Figure 2), it does not efficiently address CD8^+^ T cell activation, which is required for defense against intracellular bacteria. For example, immunization with inactivated *R. rickettsii*, the causative agent of Rocky Mountain Spotted Fever (RMSF), does not completely prevent infection and disease, although it leads to antibody production and a milder course of disease in humans [69,70]. Similarly, vaccination of US soldiers with inactivated *R. prowazekii*, the causative agent of epidemic typhus (also called war plague), during World War II ameliorated but did not prevent disease [28].

In contrast to that, WCA immunization is efficiently used for vaccination against the infection with extracellular bacteria. An example is an older cholera vaccine that consisted of dead *V. cholera*. Immunity and protection induced by this vaccine, however, was not very long-lasting and was only protective in adults [25,71]. This vaccine is no longer in use. The most widely used cholera vaccine today (Dukoral) combines a mixture of killed *V. cholera* O1 bacteria with the recombinant B-subunit of cholera toxin (CTB) [24]. Another vaccine (Shanchol) that consists of killed *V. cholera* of both serotype O1 and O139 has been recently prequalified by the world health organization (WHO) [25]. Finally, an inactivated WCA vaccine against *C. burnetii*, the causative agent of Q fever, has been developed (Q-Vax). Q-Vax is the only commercially available vaccine against Q fever today. It can provide lifelong immunity to the pathogen, but allergic reactions to the vaccine are not uncommon [26].

In addition, new approaches for the preparation of WCA vaccines are under investigation. Very recently, a new method for the preparation of a dead cell experimental vaccine against a pathogenic strain of *E. coli* has been described. *E. coli* bacteria were mineralized with a metal–organic framework that encapsulates and kills the bacteria. This framework further forms a kind of depot for the slow release of bacterial antigens, mimicking a persistent infection. This leads to the prolonged and enhanced production of antibodies in a murine infection model and enhances the survival of the animals compared to immunization with standard inactivated bacteria [72]. This method is interesting and may be applicable especially for immunization against other extracellular bacteria. WCA, however, has been shown to be not very efficient for vaccination against intracellular bacteria. One reason may be the inefficient induction of cytotoxic CD8^+^ T cells with this method.

### 2.2. Live Attenuated Bactericidal Vaccines (LAVs)

LAVs are a more promising tool than WCA for vaccination, especially against intracellular pathogens. LAVs are microorganisms that have lost pathogenicity but still have the capacity for transient intracellular replication where bacterial antigens are more likely accessible for the MHCI presentation pathway and the induction of CD8^+^ T cell responses. Loss of pathogenicity can often be achieved by growing bacteria (or viruses) under unnatural conditions for a longer period of time. This induces mutants that replicate better under these unnatural circumstances than in a natural host. Examples of such vaccines are the attenuated *M. bovis* strain Bacillus Calmette–Guerin (BCG), live attenuated vaccines against *S. enterica* ssp. [30], the only currently licensed vaccine against *B. anthracis* (Anthrax Vaccine Adsorbed (AVA) or BioThrax), and an attenuated strain of *F. tularensis* (LVS) that is available for those at enhanced risk of infection [31]. In addition, low-pathogenic strains of *O. tsutsugamushi* and *R. prowazekii*, the causative agents of Scrub typhus and epidemic typhus, have been used experimentally. The *R. prowazekii* strain Madrid E lost pathogenicity through long-term passage through embryonated chicken eggs. It possesses a mutation in the gene encoding for the methyltransferase that mediates methylation of surface proteins, including outer membrane protein B (OmpB). Hypomethylation of OmpB and other surface molecules that are involved in bacterial uptake into target cells results in reduced bacterial entry [73]. Both low pathogenic *O. tsutsugamushi* and *R. prowazekii* successfully induced immunity in humans [32,33,34]. The *R. prowazekii* Madrid E strain, however, has also been shown to become pathogenic again (*R. prowazekii* Evir) [74]. Reversion to pathogenic forms may be a general risk of using such naturally occurring apathogenic bacteria. 

A safer way can be stable genetic manipulation of bacterial pathogens for vaccination. If pathogenicity factors are known, these can be actively eliminated or mutated to become inactive variants. One example is a licensed new generation live oral cholera vaccine (Vaxchora). For this vaccine, the gene encoding for the toxigenic A subunit of cholera toxin has been deleted. In this way the bacteria exclusively express the non-toxigenic B subunit, which converts the pathogen into a non-toxic attenuated bacterial version (strain CVD103HgR) [75,76,77]. Examples of other genetically manipulated bacterial live vaccines are rare because virulence factors are often unknown. Nonetheless, there are some experimental examples. The deletion of the gene encoding for phospholipase D of *R. prowazekii*, which is involved in escape from the phagosome [78], results in an attenuated strain [79], and vaccination of guinea pigs with this strain protected the animals from lethal challenge [79]. In addition, an attenuated strain of *R. rickettsii* was generated by knockout of the OmpA protein, which, together with OmpB, mediates bacterial adherence to target cells. Immunization of guinea pigs with this strain, however, did not protect the animals from the infection [80].

### 2.3. Live Recombinant Bacteria

Live attenuated or non-pathogenic genetically manipulated bacteria represent an interesting platform for vaccine development with different strategies: (i) the use as bacterial vectors for the delivery of DNA encoding for heterologous antigens that are then expressed by eukaryotic cells as described later (please read Section 2.9.4 Bacterial Vectors), and (ii) recombinant bacteria that express foreign antigens themselves and, thus, deliver heterologous protein. Examples of the latter are described in the following.

A very recent and promising example is a new vaccine against tuberculosis (VPM1002) that is already under investigation in clinical trials [36]. This vaccine significantly improves the BCG vaccine by replacing the urease C gene of the bacteria by LLO, which has different effects. Urease C is involved in the inhibition of the acidification of the phagosome so that its replacement results in acidification of the phagosome and phagolysosome fusion. Second, LLO forms pores in the phagosomal membrane, leading to the release of mycobacterial antigens into the cytosol. This leads to a much more efficient induction, especially of CD8^+^ T cells, but also of CD4^+^ T cells, both of which are required for defense against *M. tuberculosis* [36].

In experimental approaches, *L. monocytogenes* was used as a delivery system for foreign antigens. Recombinant *L. monocytogenes* bacteria that expressed the ovalbumin (OVA) model antigen efficiently induced T cell responses [81]. Similarly, recombinant *L. monocytogenes* that were engineered to express and secrete candidate CD8^+^ T cell antigens from *C. burnetii* induced protective CD8^+^ T cell-mediated immunity in mice [44]. 

In addition, *Mycobacterium* (*M.*) *vaccae*, an environmental mycobacterium, has been used in this way. *M. vaccae* is non-pathogenic for humans and contains per se many homologous antigens to *M. tuberculosis* similar to BCG. Heat- or irradiation-killed *M. vaccae* efficiently induce CD4^+^ T_H_1 as well as cytotoxic CD8^+^ T cells in mice, and these T cells are reactive to *M. tuberculosis*-infected MΦ [82,83]. In addition, *M. vaccae* was engineered for the expression of *M. tuberculosis* antigens PE35, ESXA, ESXB, Rv2346c, Rv2347c, Rv3619c, and Rv3620c and induced a T_H_1-dominated *M. tuberculosis*-specific immune response [35]. *M. vaccae* is not only an interesting vaccine candidate against tuberculosis but may also be used for vaccination against other bacterial infections. For example, *M. vaccae* was transformed with a plasmid carrying the genetic information for the expression of fragments of the OmpA protein from *R. rickettsii*. Immunization of mice with these rickettsial OmpA-expressing bacteria followed by boost immunization with the corresponding recombinant protein fragments induced rickettsia-specific IFNγ-producing T cells and mediated at least partial protection against lethal challenge with *R. conorii*, a close relative of *R. rickettsii* [37].

Finally, some bacterial species can be used for active and direct introduction of recombinant protein antigens into the cytosol of target cells and, thus, into the MHCI presentation pathway for the induction of CD8^+^ T cells. Prototypes of these bacteria are *Yersinia* and *Salmonella*, which use the T3SS translocation system to actively export proteins into the cytosol of target cells during intracellular life and replication. Examples of exported proteins are the SspH2 protein from *Salmonella* and the Yop proteins from *Yersinia*. SspH2 or Yop proteins can be expressed as fusion proteins together with heterologous antigens in engineered *Yersinia* or *Salmonella* strains. The chimeric proteins will then be introduced into the cytosol of infected cells. In this way, protective CD8^+^ T cell responses directed against the heterologous antigen can be efficiently induced. For example, immunization with recombinant *Y. enterolitica* that expresses a fusion protein of the N-terminal region of YopE with the p60 antigen from *L. monocytogenes* results in cytosolic location of the protein, MHCI presentation, and activation of p60-specific CD8^+^ T cells [84]. Similarly, immunization of mice with engineered *Y. pseudotuberculosis* that express a fusion protein of YopE and LLO from *Listeria* results in the induction of LLO-specific CD4^+^ as well as CD8^+^ T cells [42].

In addition, *Salmonella*, which efficiently invades phagocytes such as MØ that can serve as APC as well as non-phagocytic cells [85], has been used in a similar way for experimental vaccination. An attenuated strain of *S.* Typhimurium expressing a fusion protein of the SspH2 protein from *Salmonella* and the p60 antigen from *L. monocytogenes* induced robust CD8^+^ T cell and CD4^+^ T cell responses in mice that were orally vaccinated with the recombinant bacteria [38]. In addition, attenuated *S.* Typhimurium transformed with plasmids for the expression of a nonhemolytic variant of LLO were used for oral vaccination of mice and induced excellent CD4^+^ and CD8^+^ T cell responses in addition to antibody production. Furthermore, the immunization conferred protection against lethal challenge [39]. The authors showed that the DNA was transferred to APCs for gene expression and T cell induction, although the SspH2 export system was not used in this early work.

The use of YopE from *Yersinia* for heterologous antigen delivery has been further adapted to the T3SS translocation system of *Salmonella*. Recombinant attenuated *Salmonella* Typhimurium expressing fusion proteins of YopE and LLO or p60 from *L. monocytogenes* allow for the cytosolic delivery of chimeric proteins, leading to efficient MHCI presentation. Furthermore, mice orally vaccinated with these bacteria developed high numbers of LLO- and p60-specific IFNγ-producing CD8^+^ T cells that protected the animals from challenge [40,41]. Similarly, *S.* Typhimurium engineered for the expression of virulence factors SaEsxA and SaEsxB from *S. aureus* have been recently shown to efficiently induce *S. aureus*-specific humoral and cellular immune responses with a bias to T_H_1/T_H_17 in mice upon oral vaccination and to confer protection against *S. aureus* [43]. The authors showed that the proteins were effectively translocated into the cytosol of infected MØ, so that it can be assumed that CD8^+^ T cells are activated by the vaccine and contribute to protection.

The use of live attenuated facultative intracellular bacterial vectors, e.g., *L. monocytogenes*, is especially interesting for vaccination against obligate intracellular bacteria, as these vectors can be manipulated to secrete recombinant antigens into the cytosol of infected cells for the presentation by MHCI and CD8^+^ T cell activation. The use of live recombinant bacterial vectors, as most of the other vaccination strategies, however, generally requires knowledge of the antigens that can elicit T cell responses. These are still largely unknown for obligate intracellular bacteria.

Generally, employing live attenuated live bacterial vaccines bears risks, including the possible conversion to pathogenic forms, as is known for some mutant strains of *L. monocytogenes*, and the immunoreactivity of the bacterial vectors themselves can have beneficial adjuvant effects but also may lead to non-beneficial inflammatory side effects.

### 2.4. Bacterial Ghosts (BGs)

BGs are envelopes of Gram-negative bacteria that have lost their cellular content and do not possess nucleic acids, ribosomes, or other components [86]. A currently used method for the generation of BGs is the expression of lysis gene E from synthetic enterobacteria phage KleenX174 (accession number MF426914.1) in Gram-negative bacteria. The protein forms pores in the bacterial membrane, which leads to the subsequent release of cellular contents [87,88]. In this way, BGs from several Gram-negative bacteria have been successfully prepared, including *S.* Typhimurium [89], *S. enteritides* [54], *V. cholerae* [90], *H. pylori* [91], *H. influenzae* [92], *Brucella* [93], and others. Other genes than lysis gene E have also been successfully used for the generation of BGs. For example, BGs could be prepared from *E. coli*, *Acinetobacter calcoacetate*, and *Pseudomonas stutzeri* by the cloning of plasmid pDKL02 and expression of lysis genes S, R, and Rz [94]. Peptidoglycan-free BGs generated from *Y. pestis* were revealed to be protective against infection in a murine infection model [53]. Furthermore, BGs from *S. enteritides* that were manipulated to carry surface flagellin from *S.* Typhimurium induced high antibody production in chicken [54].

An advantage of BGs is that the particles maintain all cell surface properties of the intact original bacteria. Another is that BGs can not only be manipulated for the transfer of proteins but also nucleic acids and other molecules [95]. BGs from *S. enteritides* carrying DNA encoding for different *N. ghonorheae* antigens have been shown to induce humoral and cellular immunity in mice after co-administration [55].

The methods of the generation of BGs may be especially suitable for extracellular bacteria that can be manipulated much easier than intracellular bacteria. Nonetheless, although the preparation of BGs from obligate intracellular bacteria appears to be difficult, BGs could be useful carriers of antigenic structures from these pathogens and generally represent interesting antigen delivery systems [96].

A drawback of the system may be that the so far described plasmids are not applicable to all Gram-negative bacteria, that plasmids may get lost, and that the co-transfer of a resistance gene for selection may be laterally transferred, which is also true for recombinant live bacteria.

### 2.5. Outer Membrane Vesicles (OMVs)

Gram-negative bacteria release membrane vesicles from the bacterial cell wall into the environment. These are frequently called OMVs. OMVs improve bacterial survival by different mechanisms including the neutralization of antimicrobial peptides, disposal of bacterial waste such as misfolded and stress molecules, gene transfer, transmission of virulence factors into host cells, protection against phages, and, last but not least, modulation of the immune response [97].

OMVs carry many bacterial antigens and preserve features that are identical to the bacterial membrane. These include outer membrane proteins that are anchored in the lipid membrane and can serve as antigens for the recognition by antibodies; they transport antigens that have the potential to elicit T cell responses, including CD8^+^ T cell responses, when transferred into the cytosol of target cells, and they carry pathogen-associated molecular patterns (PAMPs) such as lipopolysaccharide (LPS) that are capable of stimulating innate immune responses that are necessary for efficient induction of T cell responses. These features make OMVs an interesting tool for vaccination, especially against intracellular bacteria where T cell responses are needed for host defense. OMVs have been investigated for more than 20 years and can be prepared by different methods: (i) isolation of vesicles that are spontaneously released by the bacteria, (ii) application of a detergent such as deoxycholate that is also used to detoxify and reduce amounts of LPS that could be harmful in high concentrations when administered to humans [51], and (iii) detergent-free methods such as sonication [52]. Generally, detergents may alter molecule structures and epitopes and, thus, immune reactions. 

Today there are four licensed vaccines available that are based on OMVs. All of these are directed against *N. meningitidis* serogroup B bacteria (Bexsero/4CMenB, VA-MENGOC-BC, MenBVac, MeNZB) [51]. In contrast to *N. meningitidis* serogroups A, C, W, and Y, against which polysaccharide conjugate vaccines are effective, the development of vaccines against *N. meningitidis* serogroup B has been challenging because the capsular polysaccharide from these bacteria is poorly immunogenic and shows homologies to fetal neural tissue [98]. Major immunogenic determinants of OMV vaccines against *N. meningitidis* are the outer membrane proteins Porin A (PorA), Neisseria heparin binding antigen (NHBA), human F factor binding protein (fHbp), and Neisseria adhesin A (NadA). While PorA has been described as an immunodominant antigen before [99], the other three components were identified by reverse vaccinology [100]. The first OMV vaccines (MenBVac, VA-MENGOC-BC) were developed already in the 1980s, and another one (MeNZB) was produced in the late 1990s. Of these strain-specific vaccines, only VA-MENGOC-BC and MeNZB have been used for vaccination campaigns in Cuba and Brazil in 1989–1991 (VA-MENGOC-BC) and New Zealand in 2004–2006 (MeNZB). The first OMV vaccine to confer broad protection against several serogroup B strains (Bexsero/4CMenB) was not available until 2014 [101]. Bexsero/4CMenB is a multicomponent vaccine that contains three purified recombinant proteins (NHBA, fHbp, and NadA) in addition to OMVs from the New Zealand *N. meningitides* serogroup B strain as a source of PorA. Finally, it should be mentioned that OMV vaccination against *N. meningitidis* shows some encouraging cross-reactivity against *N. gonorrhea* [52,102].

OMVs can also be used for the transfer of heterologous antigens. For example, OMVs derived from recombinant mutant *Y. pseudotuberculosis* bacteria were revealed to be efficient carriers for the delivery of a PcrV-HitA_T_ fusion antigen from *P. aeruginosa*. Immunization of mice with these OMVs resulted in protection against challenge with the pathogen, which was dependent on a robust CD4^+^ as well as CD8^+^ T cell response rather than the production of antibodies [103]. Similarly, membrane vesicles of *S.* Typhimurium were found to activate the antigen-presenting functions of DCs and MØ and to induce CD4^+^ T cell responses and antibody production in mice, resulting in significant protection of mice against challenge with live S. Typhimurium [104]. In addition, immunization of mice with OMVs from *S. flexneri* induced specific antibodies production and protected the animals from lethal challenge with the pathogen [105]. Further attempts have been undertaken to use OMVs for vaccination against *V. cholerae*, *B. pertussis*, *M. smegmatis*, BCG, *C. trachomatis*, and *T. pallidum* (syphilis) [51,52]. The use of OMVs has been further discussed for vaccination against *S. pneumoniae*, *A. baumannii,* and *K. pneumoniae* [106], as well as viral infections such as Influenza A H1N1 Virus and MERS-CoV [107]. In contrast to the mentioned bacterial pathogens, *S. aureus* releases OMVs that carry the alpha toxin Hla and induce cell death in a Hla-dependent manner [108,109]. Thus, *S. aureus*-derived OMVs as well as recombinant active Hla protein cannot be directly used for immunization. In this context, it is worth mentioning that the toxicity of the *S. aureus* Hla toxin could be neutralized by the spontaneous insertion of the toxin into PLGA nanospheres coated with red blood cell membrane. Furthermore, immunization of mice with these nanospheres induced the production of neutralizing antibodies against Hla more efficiently than the heat-inactivated toxin and more efficiently enhanced the survival of the animals after challenge with the active toxin [110].

OMVs can be most easily prepared from genetically engineered *E. coli* that expresses immunogenic antigens fused to appropriate leader sequences to be expressed in the outer membrane. For example, *C. muridarum* DO serine protease HtrA was expressed in *E. coli* fused to the OmpA leader sequence, and OMVs prepared from these bacteria induced the production of protective antibodies against HtrA in mice [111]. In addition, a hypervesiculating *Salmonella* Typhimurium strain has been described. OMVs from these bacteria that were manipulated to express an ovalbumin (OVA) fragment on the vesicle surface induced potent DC maturation and a predominantly OVA-specific CD8^+^ rather than CD4^+^ T cell response, most likely via cross-presentation mechanisms of the OVA peptide [112]. *E. coli* can further be manipulated to produce OMVs with reduced endotoxicity for safer antigen delivery, e.g., through inactivation of the MsbB (LpxM) lipid A acyltransferase, which is involved in the generation of LPS [113]. More recently, artificial lipid membrane vesicles that contain or adsorb desired antigen for delivery have also been developed, which employ natural or synthetic polymers that lack endotoxic activity [114,115,116,117].

Finally, OMVs can not only be engineered to carry the desired antigen repertoire but may also be modified to deliver antigen into certain target cells, e.g., APCs such as DCs, allowing for efficient induction of T cell responses, including CD8^+^ T cell activation. With regard to T cell activation, the self-adjuvanticity of the vesicles is an advantage. Bacterial OMVs activate APCs to express cytokines in addition to CD80 and CD86 [112,118]. These costimulatory molecules provide the essential second signal for T cell induction apart from antigen recognition through the T cell receptor.

The mechanisms of OMV entry into target cells are not well understand, and it is not yet clear how CD8^+^ T cell activation through antigen delivery via OMVs is achieved. Clathrin- and caveolin-dependent mechanisms as well as the use of lipid rafts and direct membrane fusion have been discussed [119]. The latter would result in the release of cargo into the target cell cytosol and direct introduction of antigens into the MHCI presentation pathway. In other cases, cross presentation, which can occur via fusion of endosomal vesicles with MHCI-containing vesicles or release of antigens from endosomes into the cytosol, may occur similarly to vaccination with WCA or subunit vaccines (Figure 2).

A disadvantage of OMVs is the instability of the vesicles. This problem may be overcome by coating nanoparticles with OMVs or incorporating OMVs into polymeric NPs. The enhanced stability of OMVs was demonstrated for OMVs from *E. coli* by coating them with AuNPs [120], as well as for OMVs from *S. flexneri* that were encapsulated by PA NPs, and was revealed to be highly efficient in the induction of protective immunity in mice [121].

Generally, OMVs clearly represent a promising tool for vaccination against bacterial infections, as they highly mimic the natural bacterial surface, containing several relevant antigens that can be recognized by B and T cells.

A general advantage of the use of WCA, LAV, BGs, and OMVs is that vaccination with these agents does not require knowledge of immunodominant antigens that are recognized by B and T cells, which is a prerequisite for all other vaccines.

However, direct application of these methods for vaccination against obligate intracellular bacteria is difficult. Due to the obligate intracellular lifestyle and the need for intracellular passage through eukaryotic cells, the large scale and standardized preparation of these agents is generally difficult. For the same reason, the preparation of OMVs and BGs, of which the latter requires genetic manipulation, is also a problem with regard to intracellular bacterial pathogens.

### 2.6. Toxoids and Recombinant Proteins

Toxoid vaccines are comprised of chemically or genetically inactivated exotoxins that are released by several bacteria, including *V. cholerae*, *C. diphteriae*, *C. tetani*, *B. anthracis*, *Clostridium* (*C.*) *botulinum*, *S. aureus*, *Y. pestis*, *E. coli*, *S. dysenteria*, rickettsial ssp., and *L. monocytogenes*. These exotoxins can function in different ways. The A subunits of diphtheria toxin (DTA) and *S. dysenteria* toxin (STA) are taken up by receptor-mediated mechanisms and inhibit cellular protein synthesis in different ways, leading to cell death. While DTA mediates ADP-ribosylation and inhibition of eukaryotic elongation factor 2 (eEF2) [122], STA cleaves the 28S rRNA from the 60S ribosomal subunit [123], both of which are essential for protein translation. Similar or identical toxins to STA are produced by pathogenic strains of *E. coli* (STEC) [124,125]. *E. coli* additionally releases hemolysin [126]. *B. anthracis* produces three exotoxins (protective antigen (PA), lethal factor (LF), and edema factor (EF)) that act together. PA is responsible for receptor binding and translocation of EF and LF into the cytosol of target cells [127] where these effectors exert cytotoxic effects via their enzymatic activity, acting on second messenger signaling pathways (LF, calmoduline-dependent adenylate cyclase) and map kinase (MAPK) signaling (LF, metalloprotease) [128]. Similarly, the cholera toxin subunit A (CTA) acts on second messenger signaling by constitutive activation of adenylate cyclase, while subunit B (CTB) is responsible for receptor binding. The activity of CTA leads to increased levels of cyclic Amp and as a major result to the massive efflux of water from intestinal cells [129]. The B subunits of tetanus and botulinum subunit toxin are neurotoxins that lead to proteolytic cleavage of synaptobrevin in neuronal cells, resulting in the blockage of the release excitatory neurotransmitters and of g-aminobutyric acid (GABA), which is an inhibitor of motoneurons. Overall, this leads to overactivity of motoneurons and dangerous spasms [130]. *L. monocytogenes* produces several exotoxins including listeriolysin (LLO) and phosphatidyl-specific phospholipase AC (PlcA). LLO is a pore-forming hemolysin that plays a role in phagosomal escape, which is supported by PlcA [131]. Similarly, phospholipases excreted by rickettsial ssp. have been implicated in the escape of the bacteria from the phagosome [131]. *S. aureus* releases toxic superantigens, leading to nonspecific T cell activation and hemolysins [132].

Exotoxins are present in the cytoplasm or periplasm of bacteria and are either actively excreted or released when the bacteria are damaged. For some of the mentioned pathogens, the exotoxins represent primary virulence factors that are harmful to the host and cause disease, rather than the bacteria themselves. This is clearly true for diphtheria, tetanus, and pertussis, against which toxoid vaccines are successfully in use (DTaP combined vaccine) (https://www.cdc.gov/mmwr/volumes/67/rr/pdfs/rr6702a1-H.pdf; accessed on 16 January April 2022). In addition, vaccines against the *B. anthracis* under investigation are based on toxoid immunization with PA as the target antigen (rPA102) [45,46,47,48,49], which shields the cells from the action of EF and LF and induces, in combination with an adjuvant, comparable immunity as the currently licensed attenuated live strain vaccine [133]. A botulinum toxoid vaccine (PBT) is available through the centers for disease control (CDC) as an Investigational Drug (IND), (https://blink.ucsd.edu/_files/safety-tab/research/biosafety/Botulinum_Neurotoxin_Vaccination.pdf; accessed on 10 January 2022). In the case of *S. aureus*, an experimental toxoid vaccine combining several superantigens from *S. aureus* in a fusion protein induced antibodies that were capable of neutralizing superantigen activity and protected mice from septic shock [50].

In addition to excreted exotoxins, several bacterial pathogens including *Salmonella* ssp., *Shigella* ssp., *V. cholerae*, and *Y. pestis* can directly inject toxins from the bacteria into the cytosol of target cells employing a type III secretion system [134]. In the case of *Yersinia*, multiple virulence factors, outer proteins (Yops), are introduced into the cytosol of target cells, namely YopE, YopH, YopM, YopO/YpkA, YopP/YopJ, and YopT. These molecules inhibit host immune responses by interfering with intracellular signaling pathways, such as the activation of MAPK and NF-kB signaling, that are involved in inducible cytokine and chemokine expression, block phagocytosis, and destroy the actin cytoskeleton of target cells [135,136]. At least some of these proteins represent important and dominant antigens for recognition by CD8^+^ T cells. An example is YopE. Vaccination of mice with attenuated *Y. pestis* followed by challenge with virulent bacteria leads to the generation of a high percentage of CD8^+^ T cells that specifically react to YopE [137]. Moreover, a CD8^+^ T cell epitope of YopE (YopE_69-77_) has been identified, and immunization of mice with this epitope confers protection against lethal challenge with *Y. pestis* [137]. Finally, genetically modified attenuated *L. monocytogenes* that expresses recombinant YopE from *Yersinia* or cholera toxin in addition to the YopE_69-77_ CD8^+^ T cell epitope efficiently induces CD8^+^ T cell-mediated immunity against *Y. pseudotuberculosis* in mice [138]. Thus, YopE represents a promising candidate for subunit vaccination against plague.

For *L. monocytogenes*, it was shown that experimental vaccination of mice with recombinant non-functional LLO protein together with cholera toxin protects the animals from death upon challenge with the pathogen [139]. Surprisingly, this effect was T cell-dependent and independent from antibodies that could neutralize the toxin. Thus, this potential vaccine can be considered as a subunit vaccine. For vaccination against *N. meningitidis*, a recombinant protein vaccine (Trumenba) is in use. It consists of recombinant lipidated fHbp.

Protein vaccines are commonly in use against viral infections and may be applicable for other bacterial infections too, including obligate intracellular bacteria where humoral responses are less important than cellular immune responses, especially the activation of CD8^+^ T cells. In this case, the use of liposomes may help to increase cellular immune responses compared to immunization with “naked” protein. It is well known that liposomes are internalized by APCs such as MØ and then cross-presented to cytotoxic CD8^+^ T cells [140]. Other methods that are applicable to enhance immune responses to recombinant proteins are the use of NPs (please see Section 2.8. Antigen Delivery with Nanoparticles (NPs) or BGs. Generally, subunit vaccination requires knowledge of the immunodominant antigens of the pathogen, and in the case of intracellular bacteria, especially of those that are recognized by CD8^+^ T cells. Although great progress has been made in recent years, this knowledge is still rare. Examples of antigens from obligate intracellular bacteria that are recognized by T cells are summarized in Table 2.

### 2.7. Polysaccharide Conjugate Vaccines

Polysaccharide conjugate antigens contain immunogenic antigens or antigen fragments of a pathogen. Commonly used bacterial antigens are polysaccharides that are part of the cell wall and protect the bacteria from complement activation and killing in the blood and from phagocytosis by phagocytic cells. Bacterial capsular polysaccharides are commonly used as conjugates with a carrier protein to achieve a high affinity antibody response. This response would not be induced by polysaccharides alone, as these are T cell-independent (TI) antigens that are recognized by B cells. B cells need to undergo a germinal center reaction to produce high affinity, class-switched antibodies and to produce a memory response, which requires T cell help. With conjugate vaccines, T cell activation is achieved by the recognition of antigenic peptides derived from the carrier protein [163]. Examples of bacterial polysaccharide conjugate vaccines are vaccines against *H. influenzae* type b (PedvaxHIB, ActHIB, HibTITER), pneumococci (Prevnar, Pneumovax 23), meningococci (Menactra, Menveo, Menomune), and pertussis (part of the DTaP combined immunization). Other conjugate vaccines have been described for immunization against Shigella, with one being licensed [164], *Salmonella enterica* serovar Paratyphi, and *Salmonella enterica* serovar Typhimurium, against which conjugate vaccines have been licensed in India and China and prequalified by the WHO [30].

A new method for the production of conjugate vaccines is genetically engineered *E. coli* that produces and excretes polysaccharides that are directly linked to carrier proteins from the periplasm into the environment [165]. This method facilitates large scale production and purification of such vaccines. Polysaccharide conjugate vaccines are applicable for many extracellular bacteria, as they efficiently induce antibody production.

### 2.8. Antigen Delivery with Nanoparticles (NPs)

In recent years, NPs have gained much attention for the use of antigen delivery for vaccination. These include inorganic NPs (gold, silica, iron, zinc oxide, and carbon NPs), synthetic polymeric NPs (poly(anhydride) (PA), polylactic acid (PLA), polylactic-co-glycolic acid (PLGA), and polyethylene glycol (PEG)), as well as natural polysaccharide-based polymers (e.g., chitosan).

Antigens can either be attached to the surface or encapsulated by NPs, which can enhance antigen stability and also prolong antigen release for efficient immune activation. Surface attachment of antigen, mainly inorganic NPs, leads to the presentation of repetitive epitopes that can be efficiently bound by multiple B cell receptors, resulting in B cell activation. In addition, attachment or encapsulation of antigens efficiently increases the uptake by APCs, leading to antigen presentation via MHC molecules and activation of T cells.

An advantage of inorganic NPs, although these are not biodegradable, is that synthesis can be controlled, and that the surface of these NPs can be easily modified to improve antigen attachment, e.g., coating with carbohydrates or lipids.

Gold (Au) NPs already play an important role in nanomedicine. They are suitable for several applications including vaccination and can not only provide higher immunogenicity of a vaccine but also higher storage stability. It is interesting to mention that AuNPs are able to elicit CD4^+^ as well as CD8^+^ T cell responses. For example, AuNPs conjugated to peptides from the *L. monocytogenes* LLO were efficient in the induction of T cell responses in mice and T cell-mediated protection against challenge with the pathogen [166,167,168]. Further, conjugation of the F1 antigen *Y. pestis* to AuNPs highly enhanced immunogenicity of the protein when used for the vaccination of mice [169]. Other examples of successful experimental vaccines are AuNPs conjugated with flagellin peptide for immunization against *P. aeruginosa* [170], lipopolysaccharide for immunization against *B. mallei* [171], and tetanus toxoid for vaccination against *C. tetani* [172,173]. In addition, zinc oxide nanoparticles coupled with ScaA protein from *O. tsutsugamushi* have been successfully used for the induction of protective immunity in mice [161], and silica NPs conjugated with either VirB9-1 and VirB9-2 or VirB9-1 and VirB10 from *Anaplasma* (*A.*) *marginale*) efficiently induced humoral as well as T cell responses in immunized mice [174,175].

In addition, polymeric NPs have been successfully used for experimental immunization against bacterial infections. An advantage of polymeric NPs is that these are highly biocompatible and biodegradable. Examples are the encapsulation of domain 4 from protective antigen (PA) from *B. anthracis* by PLGA nanospheres [176], coating of PLGA with red blood cell membrane followed by the insertion of the alpha toxin from *S. aureus* [177], and the encapsulation of tetanus toxoid from *C. tetani* into PLGA nanospheres [178]. Furthermore, immunization of mice with outer membrane vesicles (OMVs) from *S. flexneri* into PA NPs successfully induced protection against challenge [121]. PA NPs are also suitable for oral vaccination, e.g., efficient induction of immunity in mice against *S. enteritidis* [179]. Finally, yellow carnauba wax NPs carrying antigens from *M. tuberculosis* also induced protective immunity in mice [180,181]. 

Table 3 provides an overview of NPs used for experimental vaccination against bacterial infections. Most methods describe immunization with NPs conjugated with protein. NPs, however, are also useful for the delivery of DNA, as described later.

### 2.9. Nucleotide Immunization

#### 2.9.1. Plasmid DNA

Another part of third-generation vaccines is the use of plasmid DNA that carries the genetic information for the expression of antigenic determinants from pathogens. Depending on the design of antigenic expression in the eukaryotic target cell (secreted or cytosolic antigen), DNA vaccination addresses humoral as well as cellular CD4^+^ or CD8^+^ T cell responses. A great advantage over other kinds of vaccines is the high stability of plasmid DNA and the ease of production employing prokaryote microorganisms. The efficiency of vaccination with plasmid DNA depends on the route of injection, with intracutaneous immunization being more effective than intramuscular or subcutaneous injection [185].

DNA vaccination has been successfully applied in experimental vaccination against bacterial infections. One example is the immunization of mice with plasmid DNA encoding for hsp65 from *M. leprae*. This vaccination induced protective immunity against *M. tuberculosis* when combined with chemotherapy [56]. Another promising vaccine against *M. tuberculosis* is a plasmid designed for the expression of T cell epitopes from Esat6 and FL as an adjuvant. Similar to the use of chitosan NPs coupled with DNA encoding for T cell epitopes from Esat6 and FL, intramuscular injection of this DNA vaccine into mice followed by an intranasal boost with epitope peptides induced a pronounced CD4^+^ T_H_1 response and cytotoxic CD8^+^ T cells in addition to antibodies and was protective against challenge [57]. Successful experimental DNA vaccination approaches against other bacterial infections have also been described. A plasmid carrying the genetic information for the expression of the immunogenic part of PA from *B. anthracis* induced a mixed CD4^+^ T_H_1/T_H_2 response with the release of IFNγ and IL-4 in addition to neutralizing antibodies that were protective against lethal challenge with PA and LF toxins [58].

Protection against *L. monocytogenes* can be mediated by cytotoxic CD8^+^ T cells, and as mentioned before, an H-2K^d^-restricted CD8^+^ T cell epitope has been identified (LLO_91-99_). Most attempts at DNA vaccination have included the LLO antigen or this epitope. A plasmid encoding for the whole LLO protein was used for intramuscular immunization of BALB/c mice but failed to induce protective immunity [59]. This may be ascribed to the cytotoxic effects of this toxin. Therefore, plasmids for the expression of mutant LLO with reduced toxicity or wildtype LLO, or mutant secreted versions of LLO were constructed, of which only the secreted mutant form was protective against challenge upon immunization [59]. Another explanation for the failure of immunization with plasmid DNA encoding for whole LLO could be that the protein is not efficiently expressed due to codon usage differences between the bacteria and the eukaryotic target cells. Vaccination of BALB/c with codon-optimized plasmid DNA for the expression of LLO_91-99_ in mice induced cytotoxic CD8^+^ T cells that expressed IFNγ and conferred better protection against lethal challenge than non-codon-optimized DNA [39,60]. In addition, other epitopes from LLO have been integrated into the vaccine by the same authors, and immunity and the magnitude of protection after vaccination was clearly dependent on the level of activated cytotoxic CD8^+^ T cells [61]. These studies indicate that optimization of codon usage may be necessary for some bacterial species for optimal protein expression in target species.

In the case of *Y. pestis*, there is one description of immunization of mice with plasmid DNA encoding for the V antigen, which efficiently induced antibody production by gene gun vaccination rather than via the injection route [62]. Protection against challenge upon vaccination, however, was not followed in this study. For *Y. enterocolitica*, hsp60 has been shown to be a protective antigen that is targeted by humoral as well as cellular immune responses in mouse models of infection [186]. Immunization of mice with a plasmid encoding for this protein induced an IgG2a antibody response, arguing for a CD4^+^ T_H_1 response, antigen-specific lymphocyte proliferation, and IFNγ production, as well as protection against oral or intravenous lethal infection with the pathogen, which was dependent on both CD4^+^ and CD8^+^ T cells [63,64]. Because protection against *Yersinia* depends on a T_H_1-type response, protection against *Y. enterocolitica* by DNA vaccination was further improved by the coexpression of IFNγ linked to the expression of hsp60 via an IRES sequence [64]. Surprisingly, although hsp60 is highly conserved among bacteria, the immunization did not confer cross-protection to *Y. pseudotuberculosis* [64]. Plasmid DNA vaccination is also under investigation against many other bacterial infections, including *C. pneumoniae*, enterotoxic *E. coli*, *H. pylori*, *Leptospira interrogans*, *Pseudomonas aeruginosa*, *Borrelia burgdorferi*, *S. pneumoniae*, *S. aureus*, and *Chlamydia* ssp [31].

Apart from the clear advantages and encouraging results from experimental vaccination approaches, the use of DNA also bears risks. The same is true for viral and bacterial vectors, a major concern being the integration of the DNA into the host cell genome that is undirected and can potentially lead to host cell gene destruction, cellular damage, or tumor formation. Another is the induction of autoimmune responses. The risk of integration, however, seems to be limited and lower than the natural mutation rate [187,188,189], and only mild side effects have been observed in clinical trials [190,191,192]. In addition, a challenge is the efficient introduction of the DNA into the eukaryotic cell. Although vaccination with pure plasmid DNA has been used in experimental studies with encouraging success, superior delivery systems are needed for DNA delivery and induction of potent immune responses. A common method for the delivery of DNA (and also proteins) into eukaryotic cells is still the use of liposomes [193,194], but some modern methods of DNA transfer have also been described. Very recently, a new approach by complexation of plasmid DNA with polyethylenimine (PEI) by coprecipitation has been reported [195]. These complexes are efficiently taken up by eukaryotic cells, resulting in gene expression. Apart from that, BGs, NPs, and viral and bacterial vectors are also interesting methods for DNA delivery.

#### 2.9.2. DNA Bound to NPs

Apart from proteins, polymeric NPs have been successfully used for the transfer of DNA encoding for immunogenic bacterial determinants and experimental immunization (Table 3). One example is a plasmid encoding for the mycobacterial hsp65 protein encapsulated into PGLA NPs [184]. Immunization of mice with these NPs resulted in efficient antibody and T cell responses and protection against challenge with virulent *M. tuberculosis*. Another example is the encapsulation of a DNA encoding for T cell epitopes from mycobacterial 6 kDa early secretory antigenic target (Esat6), which is a dominant target antigen for cell-mediated immunity in the early phase of tuberculosis into chitosan NPs together with fms-like tyrosine kinase 3 ligand (FL) as an adjuvant. FL is a growth factor that promotes the growth of T cells, B cells, and DCs, and it enhances immune responses [196,197,198]. Immunization of mice with these NPs induced protective T cell responses in mice [182].

#### 2.9.3. Viral Vectors

Adenoviruses and modified vaccinia virus Ankara (MVA) are the most frequently used viral vectors for vaccination. The safety of MVA is well documented [65,199], and vectors for the vaccination against bacterial infections are in development. For example, recombinant MVA85A, expressing the 85A antigen from *M. tuberculosis*, has already undergone clinical testing for the vaccination against *M. tuberculosis* in phase I to IIb trials [65], and experimental immunization of animals, including heterologous boost immunization after BCG vaccination, results in reduced bacterial loads upon challenge with the pathogen [200,201]. In a phase IIb clinical trial in children in Africa, however, boost immunization with MVA85A following BCG vaccination was not more effective than immunization with BCG alone [202]. The vaccine might be improved by introducing additional antigens. A multivalent recombinant MVA expressing fourteen antigens representative for the different phases of *M. tuberculosis* infection has been generated, and when used for the vaccination of mice and non-human primates, the efficient induction of CD4^+^ as well as of cytotoxic CD8^+^ T cells directed against several antigens was observed [203]. More recently, the same group developed a recombinant MVA virus expressing ten antigens from *M. tuberculosis* (MVATG18598) that efficiently induced T cell-mediated immunity and antibody production against all of these antigens in different mouse strains. In addition, vaccination with MVATG18598 in combination with antibiotic therapy reduced the bacterial burden in the lungs of mice upon challenge [204].

Similarly, a new adenoviral vector, AdAg85A, for the expression of mycobacterial protein 85A, was generated some years ago. The vector was used for the transduction of DCs that were further used as cell-based vaccines for the immunization of mice, resulting in efficient induction of CD4^+^ and cytotoxic CD8^+^ T cells and protection against *M. tuberculosis* infection [205]. Furthermore, the vector has been applied in pre-clinical and phase I clinical trials with promising results [206]. The use of viral vectors for vaccination against other bacteria has not been described yet. However, viral vectors represent an interesting tool, especially for vaccination against intracellular bacteria, as they can efficiently deliver DNA into eukaryotic cells for antigen expression

#### 2.9.4. Bacterial Vectors

Employing bacteria as vectors for the transfer of DNA requires that the bacteria be transformed with plasmids that contain eukaryotic expression cassettes for the expression of the desired antigen in host target cells. Upon infection with the modified bacteria or uptake of the bacteria by host cells, these plasmids must then be introduced into the nucleus of the eukaryotic cell so that the eukaryotic transcription machinery can do its work. Depending on the design of the transgene, it can be expressed by the eukaryotic cell as a secreted extracellular protein, presented predominantly by MHCII, or as a cytosolic protein for the presentation by MHCI. In this way, humoral as well as cellular immune responses can be induced. 

A promising candidate as a recombinant live bacterial vector for DNA transfer for antigen expression is the non-pathogenic *Lactococcus* (*L.*) *lactis* bacterium. The bacteria are capable of delivering plasmid DNA into mammalian host cells, leading to transgene expression [207,208,209,210]. The capability of DNA delivery by this bacterium, however, is limited, as is the induction of cellular immune responses [211]. More efficient transfer of DNA into mammalian host cells may be achieved with naturally pathogenic bacterial vectors that have a facultative intracellular lifestyle. *Yersinia* (*Y.*) *enterolitica*, *Shigella* ssp., *Salmonella* ssp., and *L. monocytogenes* are being explored as candidate DNA delivery vectors of this category [211]. All of these bacteria are capable of delivering DNA into the cellular nucleus for gene expression via the cellular expression machinery. *Y. enterocolitica* can survive within host tissues for several days, increasing the amount of cargo DNA during replication. In addition, it can infect epithelial cells [212]. Genetically modified attenuated *Y. enterocolitica* that carries the DNA encoding for the *Brucella* (*B.*) *abortus* antigens bacterioferritin (BFR) and P39 was shown to induce *Brucella*-specific antibody production and T_H_1 responses in vaccinated mice. Furthermore, vaccination with these bacteria conferred resistance to *B. abortus* infection [66].

For the use of *Salmonella* as a bacterial vector, several attenuated strains, predominantly from *S. enterica* serovar Typhimurium, which are capable of invading host cells but incapable of replicating within the cells, have been developed [211]. As these bacteria address MØ that serve as APCs, they are good candidates to induce cellular immune responses, including CD8^+^ T cells, in addition to antibody production. Another great advantage of these bacteria is that the vaccine can be administered orally. So far, there have been only a few descriptions of *S.* Typhimurium as a vector-based vaccine against bacterial infections. One example is attenuated *S.* Typhimurium carrying plasmids with the genetic information of subunits A and B from *Helicobacter* (*H.*) *pylori* urease. Intranasal vaccination of mice with these bacteria efficiently induced CD4^+^ T_H_1 and T_H_2 responses as well as antibody production. Furthermore, around 70% of the mice were protected against challenge with *H. pylori* [67]. Attenuated *S.* Typhimurium as an oral vaccine and DNA delivery system is also under investigation for immunization against several viral infections, including SARS-CoV-2. The introduction of the genetic information of the modified spike protein from SARS-CoV-2 into *S.* Typhimurium and oral immunization of mice with these bacteria resulted in the induction of humoral as well as cellular immune responses, including the activation of cytotoxic CD8^+^ T cells [213].

Other good candidates for the use as bacterial DNA vaccine carriers are *Shigella* ssp. The bacteria are able to evade phagosomes after entry into cells, predominantly MØ, and, in contrast to *Salmonella*, replicate freely in the cytosol. This allows for effective delivery of DNA cargo into the cellular nucleus. *S. flexneri* encoding for human immunodeficiency virus 1 (HIV-1) gp120 antigen has been successfully used for intranasal immunization against infection with HIV in a murine infection model, and it induced a robust CD8^+^ T cell response when given intranasally [214]. Similar immunogenicity by intranasal vaccination was observed for *S. flexneri* carrying the DNA encoding for the gag protein of HIV-1 [215]. Because of its capability to efficiently induce cytotoxic CD8^+^ T cell responses, *S. flexneri* may also represent a vaccination tool against intracellular pathogenic bacteria. 

Another promising bacterial vector is L. monocytogenes, which may potentially be used for oral DNA vaccination because of its ability to infect intestinal epithelial cells [216,217]. Attenuated recombinant *L. monocytogenes* carrying a plasmid encoding for *M. tuberculosis* antigen 85 complex and MPB/MPT51antigen induced a robust cellular immune response in mice upon intraperitoneal vaccination [68]. In another study, however, it was observed that *L. monocytogenes* transferring a plasmid encoding for the OVA model antigen failed to induce specific T cell responses, whereas recombinant *L. monocytogenes* expressing OVA itself was successful [81].

Apart from delivering DNA into target cells, an advantage of the use of bacteria is the immunostimulatory adjuvant effect on APCs by the bacteria themselves. The same is also true for BGs. This occurs via the recognition of pathogen-associated molecular pattern (PAMPs) molecules by pattern recognition receptors (PRR) that induce the release of cytokines and enhance the antigen-presenting properties by activation of the expression of costimulatory molecules (CD80, CD86).

#### 2.9.5. mRNA

mRNA vaccines have been clearly proven to be efficient and safe methods of inducing protective immunity against viruses, being in use now for more than a year for the vaccination against SARS-CoV-2. Currently, mRNA vaccines are being developed for several other viral infections, including Cytomegalovirus (CMV), Zika, Human Metapneumovirus (hMPV), Respiratory Syncytial Virus (RSV), Influenza A, Chikungunya, and Rabies virus [218]. mRNA can be delivered into eukaryotic cells by different systems, including liposomes that also may exert an adjuvant effect. These techniques are reviewed in more detail elsewhere [218]. The mRNA itself generally consists of structures that mimic endogenous mRNA in all its features. Similar to DNA, the part of synthetic mRNA that encodes a pathogen-derived antigen can be optimized for high-efficient gene expression in eukaryotic cells by codon optimization. Expression can be further maximized by the introduction of modified nucleosides such as pseudouridine and other nucleoside analogues that are common in native mRNAs [219]. The introduction of these nucleosides avoids the recognition by PRR [220] such as Toll-like receptor (TLR) 3, 7, and 8. These receptors recognize unmodified mRNA, which is common to viruses and induces the expression of IL-6 and other proinflammatory cytokines, including type I interferons IFNα/IFNβ. These type I interferons inhibit mRNA translation by activating inhibitors of translation as well as enzymes that are involved in mRNA degradation [221]. Immunological defense against viruses usually requires efficient induction of cytotoxic CD8^+^ T cells. This response is clearly addressed by mRNA vaccines. mRNAs are taken up by APCs and enter the cytosol after escape from the endosome. In the cytosol, the mRNA is translated into antigenic cytosolic protein that can be degraded by the proteasome for the presentation by MHCI and the activation of cytotoxic CD8^+^ T cells. Antigens that are secreted by the cells can be recognized by B cells and are taken up by other APCs for the presentation by MHCII and the induction of CD4^+^ T cells. These in turn assist B cells to undergo germinal center reaction to produce high affinity IgG antibodies and to build a memory response. In this way, mRNA vaccination can induce all immunological components that are also necessary for defense against other intracellular pathogens, including bacteria. mRNA, thus, is a promising tool for the adaption to vaccination against other microorganisms than viruses. So far, there have been no descriptions for mRNA vaccines against bacterial infections. As mentioned above, most vaccines that are presently developed are directed against viral infections. Nonetheless, encouraging reports exist for new experimental mRNA vaccines against *Plasmodium*, an intracellular unicellular eukaryotic parasite and the causative agent of malaria. *Plasmodium* secretes a cytokine called macrophage migrating inhibitory factor (PMIF) that prevents the development of T cell long-term memory [222]. An experimental mRNA vaccine encoding PMIF improved the induction of T helper cells and memory development and elicited *Plasmodium*-specific IgG antibodies [223]. Moreover, the T cells induced by this vaccination were revealed to be protective for unvaccinated mice against challenge with *Plasmodium* sporozoites [223]. In a second study, another protein from *Plasmodium falciparum* was used as a target for mRNA expression (*Plasmodium* (*P.*) *falciparum* glutamic-acid-rich protein (PfGARP)). During infection, this protein is presented on the surface of infected erythrocytes, and antibodies that bind to this protein kill trophozoite-infected erythrocytes by inducing programmed cell death in the parasites [224]. An mRNA vaccine encoding for this protein antigen reduced parasitemia in Aotus monkeys after infection with *P. falciparum* [224].

These encouraging observations suggest that future investigations should also focus on the development of mRNA vaccines against bacterial infectious diseases, especially those that are caused by obligate intracellular bacteria.

## 3. Conclusions

Vaccination against bacterial infectious diseases, especially those caused by obligate intracellular bacteria, remains challenging due to specific immune requirements for defense and protection, in particular the activation of cytotoxic CD8^+^ T cells. Several third-generation vaccination strategies are now available that may represent promising tools for vaccination against these pathogens as well. However, the protective immune mechanisms against various bacterial pathogens are still poorly understood and need further investigation. In particular, the immunogenic determinants of these complex pathogens are largely unknown and urgently need to be identified as a prerequisite for vaccine development. This requires appropriate animal models that can have a major impact on infection and should resemble human infection. Finally, the selection of appropriate adjuvants described elsewhere in the literature is crucial for immunization success.

## Figures and Tables

**Table 1:** Applied and experimental vaccines against bacterial infections.

Vaccine	E	C	L	Example
WCA			X	*V. cholerae* (Dukoral, Shanchol) [24,25]
			X	*C. burnetii* (Q-Vax) [26]
	X			*R. rickettsii* [27,28,29]
	X			*R. prowazekii* [28,29] (was used for the immunization of soldiers during the First World War)
	X			*O. tsutsugamushi* [28,29]
LAV			X	*M. tuberculosis* (BCG)
			X	S. *enterica* ssp. [30]
			X	*B. anthracis* (BioThrax)
			X	*F. tularensis* (LVS) [31]
			X	*V. cholerae* (Vaxchora)
	X			*O. tsutsugamushi* [32,33,34]
	X			*R. prowazekii* [32,33,34]
Live recombinant bacteria	X			*M. tuberculosis* (*M. vaccae* expressing *M. tuberculosis* antigens) [35]
		X		*M. tuberculosis* (VPM1002; BCG with urease C replaced by LLO from *L. monocytogenes*) [36]
	X			*R. rickettsii* (*M. vaccae* expressing OmpA fragments from *R. rickettsii*) [37]
	X			*L. monocytogenes* (*S.* Typhimurium expressing a fusion protein of *Salmonella* SspH2 and p60 antigen from *L. monocytogenes*) [38]
	X			*L. monocytogenes* (*S.* Typhimurium transferring DNA encoding for a nonhemolytic LLO variant) [39]
	X			*L. monocytogenes* (*S*. Typhimurium expressing fusion proteins of YopE and LLO or p60) [40,41]
	X			*L. monocytogenes* (*Y. pseudotuberculosis* expressing a fusion protein of YopE and LLO) [42]
	X			*S. aureus* (*S.* Typhimurium expressing SaEsxA and SaEsxB from *S. aureus*) [43]
	X			*C. burnetii* (*L. monocytogenes* expressing *C. burnetii* CD8^+^ T cell antigens) [44]
Toxoid/subunit vaccines			X	*C. diphteriae* (dTAP combined vaccine)
			X	*C. tetani* (dTAP combined vaccine)
			X	*B. pertussis* (dTAP combined vaccine)
			X	*N. meningitidis* (Trumenba)
		X		*B. anthracis* (rPA102) [45,46,47,48,49]
	X			*S. aureus* [50]
Polysaccharide conjugates			X	*H. influenzae*: PedvaxHIB, ActHIB, HibTITER
			X	*S. pneumoniae*: Prevnar, Pneumovax 23
			X	*N. meningitidis*: Menactra, Menveo, Menomune
OMVs			X	*N. meningitidis* serogroup B (Bexsero/4CMenB, VA-MENGOC-BC, MeNZB, MenBVac)
	X			*V. cholerae* [51,52]
	X			*B. pertussis* [51,52]
	X			*M. smegmatis* [51,52]
	X			*BCG* [51,52]
	X			*C. trachomatis* [51,52]
	X			*T. pallidum* [51,52]
BGs	X X X			*Y. pestis* [53] *S.* Typhimurium (*S. enteritides* BGs expressing flagellin) [54]) *N. ghonorhea* (*S. enteritides* BGs carrying DNA for *N. ghonorhea* antigens) [55])
Plasmid DNA	X			*M. tuberculosis* (hsp65 from *M. leprae*) [56], Esat6 T cell epitopes) [57] *B. anthracis* (PA antigen) [58]
	X			*L. monocytogenes* (LLO or LLO91-99 CD8^+^ T cell epitope) [39,59,60,61]
	X			*Y. pestis* (V antigen) [62]
	X			*Y. enterocolitica* (hsp60+/−IFNγ coexpression) [63,64]
	X			*C. pneumonia*, enterotoxic *E. coli*, *H. pylori*, *L. interrogans*, *P. aeruginosa*, *B. burgdorferi*, *S. pneumoniae*, *S. aureus*, *Chlamydia* ssp. [31]
Viral vectors		X		*M. tuberculosis* (85A antigen) [65]
Bacterial vectors	X			*B. abortus* (*Y. enterolica* encoding bacterioferritin) [66]
	X			*H. pylori* (S. Typhimurium encoding urease A and B subunits [67]
	X			*M. tuberculosis* (*L. monocytogenes* encoding antigen 85 complex and MPB7MpT51 antigen) [68]
NPs	X			please see Section 2.8. Antigen delivery with nanoparticles (NPs)

X labels the status of vaccine development and existence (E: experimental, C: in preclinical and clinical trials, L: licensed and applied).

**Table 2:** A selection of T cell antigens from obligate intracellular bacteria that have been described in the literature.

Pathogen	Antigen	Ref.
*Chlamydia*	Immunodominant CD8^+^ T cell antigens: CT529, CT511, CT461 (*C. trachomatis*)	[141]
*Anaplasma*	VirB9-1, VirB9-2, VirB10, conjugal transfer protein (CTP) (*A. marginale*)	[142,143]
*Ehrlichia*	CD8^+^ T cell antigens: Erum0660, Erum2330, Erum2540, Erum2580, Erum5000 (*E. ruminantum*) CD4^+^ T cell antigen: OMP-19 (*E. muris*, *E. chaffeensis*)	[144,145]
*Rickettsia* ssp.	SFG rickettsiae: OmpA, OmpB, Adr2, YbgF TG rickettsiae: OmpB, CD8^+^ T cell antigens: RP403, RP598 RP739, RP778	[37,146,147,148,149,150,151,152]
*Orientia*	Sta22, Sta56, ScaA	[153,154,155,156,157,158,159,160,161]
*Coxiella*	CD8^+^ T cell antigens: 17 T4SS-related proteins (*C. burnetii*) CD4^+^ T cell antigens: CBU_1835/protoporphyrinogen oxidase, CBU_1513/protoporphyrinogen oxidase, CBU_1398/SucB, CBU_0718, CBU_0307/outer membrane protein	[44,162]

**Table 3:** Antigen and DNA delivery with NPs in experimental vaccination against bacterial infections.

NP Carrier	Bacterium	Antigen	Ref.
AuNPs and chitosan-functionalized AuNPs (CsAuNPs)	*L. monocytogenes*	Listeriolysin peptide LLO_91-99_ or glyceraldehyde-3-phosphate dehydrogenase (GAPDH)_1-22_ peptide	[166,167,168]
	*Y. pestis*	F1 antigen	[169]
	*B. mallei*	Lipopolysaccharide	[171]
	*P. aeruginosa*	Flagellin peptide 1-161 (Flagellin_1-161_)	[170]
	*C. tetani*	Tetanus toxoid	[172,173]
	*E. coli*	Bacterial OMVs	[120]
Zinc oxide NPs	*O. tsutsugamushi*	ScaA protein	[161]
Silica NPs	*A. marginale*	VirB9-1, VirB9-2, and VirB10	[174,175]
Chitosan NPs	*M. tuberculosis*	DNA encoding for T cell epitopes from Esat6 and FL	[182]
	*M. tuberculosis*	Mycobacterial lipids	[183]
PLGA nanospheres	*M. tuberculosis*	Plasmid DNA encoding for mycobacterial hsp65	[184]
	*B. anthracis*	Protective antigen (PA) domain 4	[176]
	*S. aureus*	Red blood cell membrane and insertion of the alpha toxin (α-hemolysin (Hlα)) into the membrane	[177]
	*C. tetani*	Tetanus toxoid	[178]
PA NPs	*S. flexneri*	Encapsulated bacterial OMVs	[121]
	*S. enteritidis*	Flagellin	[179]
Yellow carnauba wax NPs	*M. tuberculosis*	Fusion protein of Acr, Ag85B, and heparin-binding hemagglutinin adhesion antigen (HBHA)	[181]
	*M. tuberculosis*	Ag85B and HBHA	[180]

## Data Availability

Not applicable.

## Supplementary Materials

The following supporting information can be downloaded at: https://www.mdpi.com/article/10.3390/vaccines10050751/s1. Table S1: Overview on extracellular, facultative and obligate intracellular bacterial pathogens, diseases and available vaccines; Table S2: Antibiotic resistant bacterial infections in the U.S. reported by the CDC.

## Abbreviations

Antigen-presenting cell (APC), bacterial ghosts (BGs), dendritic cell (DC), endothelial cell (EC), epithelial cell (EPC), live attenuated bacterial vaccines (LAV), macrophage (MØ), nanoparticles (NPs), outer membrane vesicles (OMVs), whole cell antigen (WCA).
