# Supplementary material for: Vaccination against Bacterial Infections: Challenges, Progress, and New Approaches with a Focus on Intracellular Bacteria
Source: Vaccines (Basel). 2022 May 10;10(5):751. doi: 10.3390/vaccines10050751 (PMC9144739; doi:10.3390/vaccines10050751)
Supplement: Supplementary file 1 [file vaccines-10-00751-s001.zip › vaccines-1682393-supplementary.pdf]

Supplementary Table S1. Overview on extracellular, facultative and obligate intracellular bacterial pathogens, diseases and available vaccines

Extracellular bacterial pathogens		
**Pathogen**	**Transmission, Disease and Symptoms, Prevalence**	**Vaccine**
Escherichia coli virulent strains (e.g. *E. coli* O157:H7 (STEC), *E. coli* (UPEC), *E. coli* (ETEC), *E. coli* O104:H4 (EHEC))	**Transmission:** contaminated food **Disease and symptoms:** gastroenteritis, hemorrhagic colitis, Crohn´s disease, urinary tract infections, neonatal meningitis, abdominal cramps, diarrhea, vomiting **Prevalence:** sporadic outbreaks	
Acinetobacter baumannii	**Transmission:** predominantly airborne (ventilator-associated infection) **Disease and symptoms:** range of different diseases, e.g. pneumonia, bloodstream infections, meningitis, wound infections, urinary tract infections, affects predominantly immunocompromised individuals **Prevalence:** occurs worldwide, estimated incidence of 1.000.000 cases per year, 50% of which are Carbapenem-resistant cases; most infections are reported from the Mediterranean region [1]	
Pseudomonas aeruginosa	**Transmission:** contaminated water, soil; in clinics contaminated hands and gloves from clinicians **Disease and symptoms:** infection of urinary tract, respiratory system, dermis, soft tissue, grastrointestine, systemic distribution via the blood and various sepsis syndromes, affects predominantly immunocompromised individuals, multidrug-resistant **Prevalence:** in 2017 32.600 estimated cases in hospitalized patients in the U.S. with 2.700 estimated deaths (https://www.cdc.gov/drugresistance/pdf/threats-report/pseudomonas-aeruginosa-508.pdf)	
Clostridioides difficile	**Transmission:** contaminated water and food **Disease and symptoms:** diarrhea, dehydration, abdominal pain, loss of appetite, nausea; reoccurrence of disease in about 20-30% of patients **Prevalence:** 0.5 million infections and 29 000 deaths estimated in 2012 in the U.S. [2]	
Vibrio cholerae	**Transmission:** contaminated water and food **Disease and symptoms: Cholera**, symptoms include diarrhea, vomiting, low blood pressure, muscle cramps, rapid heart rate, kidney failure and coma **Prevalence:** most epidemics occur in the hospital setting and in long-term care facilities, e.g., appr. 100.000 infections in hospitalized patients per year in the U.S. [3]	Vaxchora, Dukoral
Corynebacterium diphteriae	**Transmission:** droplets **Disease and symptoms: Diphteria**, symptoms include breathing weakness and fever, toxins can damage kidneys, nerve and heart **Prevalence:** in 2017 8.819 cases of diphteria were reported worldwide [4]	several combined vaccines against tetanus (e.g. Td-pur, Td-RIX), pertussis and viral pathogens
Clostridium tetani	**Transmission:** wounds contaminated with dirt, feces or saliva **Disease and symptoms: Tetanus**, toxins affect the nervous system, muscle cramps throughout the body, trouble swallowing, seizures, headache, fever, fast heart rate **Prevalence:** rare today due to effectiveness of vaccines	Te Anatoxal Berna, Tetanol and several combined vaccines

Treponema pallidum	ssp. *pallidum*	**Transmission:** sexual<br>**Disease and symptoms: Syphilis**, occurs in phases, primary symptoms include ulcer at entry site, joint, muscle and bone pain; among secondary symptoms are fever, fatigue, exanthema, lymphadenopathy; latent persistence and disseminated reoccurrence, abortion, stillbirth or harm to the child<br>**Prevalence:** in 2016 6 million infections estimated worldwide (https://apps.who.int/iris/bitstream/handle/10665/277258/9789241565691-eng.pdf?sequence=5&isAllowed=y)	
	ssp. *endemicum*	**Transmission:** skin contact, mouth-to-mouth by kissing of use of contaminated drinking vessels<br>**Disease and symptoms: Bejel** (extragenital endemic syphilis), symptoms include lesions on limbs and trunk, leg bone inflammation, gummas of the nose and soft palate<br>**Prevalence:** common in dry, hot climates especially in the countries of the eastern Mediterranean region and in Saharan West Africa	
	ssp. *pertenue*	**Transmission:** similar to ssp. *endemicum*<br>**Disease and symptoms: Frambosis**, occurs in phases, primary symptoms include itchy papule or painless ulcer on the lower leg, face (children) or breast (nursing women); generalized papules in secondary stage; latent tertiary state is symptom-free; reoccurrence in fourth stage (joint and bone deformation, cleft lip, saddle nose)<br>**Prevalence:** similar to ssp. *endemicum*	
Mycoplasma genitalium		**Transmission:** sexual<br>**Disease and symptoms:** urethritis (40% of persistent or recurrent urethritis), associated with cervicitis<br>**Prevalence:** ranges from 0.4% in young adults in the U.S. to 4.5% in The Netherlands (https://www.infectiousdiseaseadvisor.com/home/topics/sexually-transmitted-diseases/mycoplasma-genitalium-challenges-in-diagnosis-and-treatment/), makes up 15-20% of non-gonococcal urethritis (NGU) and 20-25 of nonchlamydial NGU, 40% of persistent or recurrent urethritis (https://www.cdc.gov/std/treatment-guidelines/mycoplasmagenitalium.htm)	
Borrelia burgdorferi		**Transmission:** ticks<br>**Disease and symptoms:** Lyme disease/borreliosis erythema migrans around entry site (early), disseminated infection can affect the nervous system (10% of cases), the joints, the skin and/or the heart<br>**Prevalence:** common in the northern hemosphere, e.g., 35.000 cases reported in 2010 in Europe (https://www.euro.who.int/__data/assets/pdf_file/0008/246167/Fact-sheet-Lyme-borreliosis-Eng.pdf)	

Facultative intracellular bacterial pathogens				
**Pathogen**		**Target cells**	**Transmission, Disease and Symptoms, Prevalence**	**Vaccine**
Mycobacterium	*tuberculosis*	MØ [5]	**Transmission:** airborne, droplets (person to person)<br>**Disease and symptoms: Tuberculosis**, lung infection, symptoms include (bloody) cough, fever, weight loss, fatigue, weakness; latent infection without symptoms<br>**Prevalence:** in 2020 appr. 10 million people infected worldwide with 1.5 million deaths (https://www.who.int/news-room/fact-sheets/detail/tuberculosis)	BCG
	leprae	skin MØ [6], Schwann cells [7]	**Transmission:** droplets<br>**Disease and symptoms: Leprosy**, tuberculoid form affects skin and nerves, among symptoms loss of tactile sense, myasthenia, muscle degeneration, paralysis; lepromatous form with high bacteremia and contagiousness, bacteria in the blood, lymphatic system and nerve tissue, knots and spots in the skin, ulcerative degeneration of bones, muscles and tendons, infection of inner organs, death through secondary infection rather than the pathogen itself<br>**Prevalence:** 129.389 cases on treatment in 2020 worldwide (https://www.who.int/news-room/fact-sheets/detail/leprosy)	
Tropheryma whipplei		MØ [8]	**Transmission:** fecal-oral<br>**Disease and symptoms: Whipples disease**, chronic disease, symptoms include diarrhea, abdominal pain, weight loss, joint pains, low-grade fever, neurological symptoms (e.g. dementia, memory loss, confusion), peripheral edema, hyperpigmentation of the skin, uveitis, endocarditis, pneumonia<br>**Prevalence:** in the U.S. 9.8 cases per million [9]	
Haemophilus influenzae type b*		MØ and EPCs in the lung [10]	**Transmission:** droplets<br>**Disease and symptoms:** fever, acute bronchitis, pneumonia, invasive infections with sepsis, pericarditis, meningitis, unconsciousness, apnoe, spasms, long-term symptoms (deafness, visual disorders, blindness, mental disability)<br>**Prevalence:** in 2019 7.130 cases in the U.S. (https://www.cdc.gov/abcs/downloads/HFLU_Surveillance_Report_2019.pdf)	MenHibrix , Hiberix, Liquid PedvaxHib, ActhHib
Neisseria	*meningitidis**	various cell types including EPCs, ECs, erythrocytes, phagocytes [11]	**Transmission:** sharing respiratory and throat secretions (saliva or spit)<br>**Disease and symptoms:** distribution in the blood and brain causing meningitis and sepsis predominantly under condition of immunosuppression<br>**Prevalence:** in 2019 371 cases in the U.S. (https://www.cdc.gov/abcs/downloads/NMEN_Surveillance_Report_2019.pdf)<br>serotypes A, B, C, W, X, and Y cause most diseases worldwide, in the U.S. predominantly B, C and Y (https://www.cdc.gov/meningococcal/about/causes-transmission.html)	several against serogroups A, C, W, Y, e.g. Mencevax ACWY, Meningitec, Menveo and others;<br>two against serogroup B with broad specificity: Trumenba, Bexsero/4CMenB

gonorrhoeae[*]		various cell types including EPCs, ECs, MØ ,neutrophils [12]	**Transmission:** sexual<br>**Disease and symptoms: Gonorrhoe**, symptoms include urethritis, purulent discharge from penis, vaginal discharge, burning with urination menstrual abnormalities, increased risk of prostate cancer, in women infertality and risk of ectopic pregnancy, disseminated infection can cause rash and joint pain, in newborns conjunctivitis which can result in blindness<br>**Prevalence:** in 2016 appr. 87 million estimated infections [13]	
Streptococcus pneumoniae[*]		MØ [14], erythrocytes [15]	**Transmission:** human-to-human via droplets<br>**Disease and symptoms:** pneumonia, sinusitis, otitis media, systemic distribution and sepsis, meningitis, fever chills, cough, headache, unconsciousness; children <5 years of age and the elderly are at enhanced risk<br>**Prevalence:** in 2019 30.300 cases with 3.250 deaths in the U.S. (https://www.cdc.gov/abcs/downloads/SPN_Surveillance_Report_2019.pdf)	Prevenar, Prevenar 13, Pneumovax 23, Synflorix, Vaxneuvance
Helicobacter pylori		Gastric cells [16]	**Transmission:** oral from person to person by saliva, vomiting, probably fecal material, and by contaminated food and water<br>**Disease and symptoms:** although most infections are asymptomatic, _H. pylori_ can cause nausea, bloating, burning pain in abdomen and loss of appetite, inflammation in stomach linings, gastric and duodenal ulcers and stomach cancer<br>**Prevalence:** 4.4 billion infected individuals estimated worldwide in 2015 [17]	
Salmonella enterica ssp. _enterica_ Serovars	Typhimurium[*]	gut EPCs (M cells), MØ [18, 19]	**Transmission:** contaminated food, contact with infected animals, contaminated water<br>**Disease and symptoms:** fever, gastroenteritis, diarrhea, abdominal pain<br>**Prevalence:** estimated 1.4 million cases and 600 deaths per year in the U.S. [20]	
	Typhi<br>Paratyphi	Neutrophils, MØ, DCs, EPCs [21, 22]	**Transmission:** contaminated food<br>**Disease and symptoms: Typhoid fever** and **Paratyphus**, symptoms include fatigue, headache, unproductive cough, diarrhea, disorder of consciousness, rash; pneumonia, meningitis, gastrointestinal ulcers and bleedings; persistence of the bacteria (5% permanent ejectors)<br>**Prevalence:** estimated 11-20 million infections worldwide with 128.000-161.000 deaths (https://www.who.int/health-topics/typhoid#tab=tab_1)	Vivotif, Typhim VI, Typhoral L, combined vaccines, e.g. Hepatyrix, ViATIM
Nocardia ssp.[*] (_N. asteroides_)		Neutrophils, MØ [23]	**Transmission:** inoculation into injured skin, inhalation<br>**Disease and symptoms: Nocardiosis**, slowly progressive pneumonia and lung abscesses, cough, dyspnea, fever; systemic spread, encephalitis and brain abscesses, various cutaneous infections<br>**Prevalence:** 500-1000 cases per year in the U.S. (https://rarediseases.org/rare-diseases/nocardiosis/)	
Brucella ssp.		MØ, trophoblast cells [24]	**Transmission:** infects cattle, swine, goats, sheep and dogs, transmission to humans through contact with infected animals, contaminated meat, unpasteurized milk and cheese from infected goats or sheep<br>**Disease and symptoms: Brucellosis**, fever, chills, lymphadenitis, granuloma in spleen, liver, and bone marrow	

			**Prevalence:** in 2017 381 confirmed cases in the EU with highest rates in southern EU Member States (Greece, Italy, Portugal) (https://www.ecdc.europa.eu/en/publications-data/brucellosis-annual-epidemiological-report-2017), *Brucella melitensis* is the most prevalent species	
Legionella pneumophila and other ssp.		alveolar monocytes, MØ, lung EPCs [25]	**Transmission:** airborne<br>**Disease and symptoms: Legionellosis/Legionnaire´s disease**, fever and chills, myalgia, dry cough, shortness of breath, headache, nausea, vomiting, diarrhea, neurological failures<br>**Prevalence:** 4.564 estimated case and 334 deaths in the U.S. in 2015 (https://www.cdc.gov/abcs/reports-findings/survreports/leg15.html)	
Yersinia	*pestis*	MØ, neutrophils [26], EPCs [27]	**Transmission:** between animals and humans by the bite of infected fleas, direct contact with infected tissues, and inhalation of infected respiratory droplets<br>**Disease and symptoms: Plague**, blister at site of infection, infection of the lung, high fever, bloody cough, kidney and liver necrosis, death through septic shock<br>**Prevalence:** 3248 reported cases worldwide from 2010-2015 with 584 deaths (https://www.who.int/news-room/fact-sheets/detail/plague)	
	enterocolitica[*]	EPCs [28]	**Transmission:** contaminated food and water<br>**Disease and symptoms: Yersiniosis**, fever, abdominal pain, gastroenteritis with diarrhea (often bloody)<br>**Prevalence:** have occurred in Australia, Finland, Japan, Norway, the United States, and Brazil [29]	
	pseudotuberculosis[*]	MØ [30]	**Transmission:** numerous animal reservoirs, contaminated food, soil and water<br>**Disease and symptoms:** similar to *Y. enterocolitica*<br>**Prevalence:** occurs worldwide, primarily observed in Russia and Japan, sporadically in Europe; large-scale outbreaks have only occurred in Canada, Finland, Russia, and Japan [31]	
Listeria monocytogenes		MØ, DCs, EPCs, hepatocytes [32]	**Transmission:** contaminated food, water and soil<br>**Disease and symptoms: Listeriosis**, symptoms include nausea, vomiting, diarrhea, fever, inflammation of pharynx and throat, urinary bladder and kidney, sepsis, abort, respiratory problems, encephalitis, meningitis<br>**Prevalence:** 0.1 to 10 cases per 1 million people per year depending on the countries and regions of the world, mortality rate of invasive listeriosis 20%–30% (https://www.who.int/news-room/fact-sheets/detail/listeriosis)	
Francisella tularensis	ssp. tularensis (Type A)<br>ssp. *holarctica* (Type B)	MØ, DCs, Neutrophils, DCs, hepatocytes, ECs, alveolar lung EPCs [33]	**Transmission:** bite of infected arthropods (usually ticks with voles, mice, squirrels and rabbits being natural reservoirs), contact with infected animal tissues or fluids, direct contact or ingestion of contaminated water, food or soil, or inhalation of aerosolized bacteria (https://www.centerforhealthsecurity.org/our-work/publications/francisella-tularensis-fact-sheet)<br>**Disease and symptoms: Tularemia** (ulceroglandular, okuloglandular, glandular, oropharyngeal, typhoid, pulmonary, intestinal, abdominal tularemia), various symptoms depending on entry site<br>**Prevalence:** the high pathogenic Type A occurs only in North America while the moderately pathogenic Type B is endemic throughout the Northern Hemisphere; 100–200 cases per year in the	

		U.S., fatality rate 5% up to 60% in pneumonic or septic cases without antibiotic treatment [34](https://www.centerforhealthsecurity.org/our-work/publications/francisella-tularensis-fact-sheet)	
*Staphyloccus aureus**	EPCs, ECs, fibroblasts, osteoblasts, keratinocytes, neutrophils [35, 36]	**Transmission:** contact with injured skin<br>**Disease and symptoms:** skin infections (crusting of the skin, furuncles, cellulitis, staphylococcal scalded skin syndrome (SSSS), abscesses), pneumonia, endocarditis, osteomyelitis, sepsis, toxic shock syndrome (TSS)<br>**Prevalence:** in 2018 3748 methicillin-sensitive *S. aureus* (MSSA) and 3.319 Methicillin-resistant *S. aureus* (MRSA) cases with 394 deaths (MSSA) and 437 deaths (MRSA) in the U.S. (https://www.cdc.gov/hai/eip/pdf/2018-MRSA-Report-508.pdf)	
Bordetella pertussis	alveolar MØ, lung EPCs [37]	**Transmission:** droplets (airways)<br>**Disease and symptoms: Pertussis** (whooping cough), primary symptoms include fever, snuff, dry irritant cough, in second stage staccato coughing attacks, vitreous mucus, vomiting; complications caused by secondary infections (e.g. *Haemophilus influenzae*)<br>**Prevalence:** in 2019 18.617 cases in the U.S. (https://www.cdc.gov/pertussis/downloads/pertuss-surv-report-2019-508.pdf)	several combined vaccines together with diphteria and tetanus, e.g. Boostrix, Covaxis, Infanrix, and vaccines against other pathogens
Burkholderia pseudomallei	alveolar MØ, various non-phagocytic cells [38]	**Transmission:** contaminated soil<br>**Disease and symptoms: Melioidosis**, various symptoms including wound infection, sepsis, abscesses in the lung and pneumonia, abscesses in spleen, liver, muscles and skin<br>**Prevalence:** occurs in tropical regions, estimated 165.000 cases of melioidosis per year worldwide with 89.000 deaths [39]	
Bacillus anthracis	alveolar MØ [40, 41]	**Transmission:** contaminated soil and contact with infected animals and their products; persists worldwide in livestock and wildlife<br>**Disease and symptoms: Anthrax**, cutaneous, pulmonary, and gastrointestinal forms, necrotic lesion at site of entry, systemic distribution, destruction of blood cells through toxins, sores on face, neck, arms or hand, fever, body aches, respiratory distress, septic shock and organ failure<br>**Prevalence:** endemic to many parts of South Europe, Asia, Africa, North and South America, and Australia [42]	Biothrax
Shigella *flexneri*, *sonnei*, *boydii*, *dysenteria*	gut EPCs, MØ [43, 44]	**Transmission:** fecal-oral<br>**Disease and symptoms: Shigellosis (**bacterial dysentery), symptoms include fever and severe diarrhea<br>**Prevalence:** 164.7 million cases estimated per year, 163.200 million of these in developing countries with 1.1 million deaths; 61% of deaths involves children <5 years [45]	

Obligate intracellular bacterial pathogens				
**Pathogen**		**Target cells**	**Transmission, Disease and Symptoms, Prevalence**	**Vaccine**
Chlamydia	*trachomatis*	mucosal non-phagocytic cells [46]	**Transmission:** sexual<br>**Disease and symptoms:** painful urination, vaginal discharge, painful sexual intercourse and bleedings between periods and after sex in women, discharge from the penis and testicular pain in men, rectal pain, discharge or bleeding upon rectal infection, eye infections (conjunctivitis) upon contact with infected body fluids<br>**Prevalence:** in 2016 estimated 127 million infections worldwide (https://apps.who.int/iris/bitstream/handle/10665/277258/9789241565691-eng.pdf?sequence=5&isAllowed=y)	
	pneumoniae		**Transmission:** droplets<br>**Disease and symptoms:** pneumonia, associated with artherosclerosis, coronary artery disease, asthmatic bronchitis, involved in multiple sclerosis<br>**Prevalence:** unknown, sporadic outbreaks (https://www.cdc.gov/pneumonia/atypical/cpneumoniae/publications.html#outbreaks)	
Anaplasma phagocytophilum		neurophils [47], ECs [48]	**Transmission:** ticks, causes granulocyte anaplasmosis in dogs (reservoir)<br>**Disease and symptoms: Human granulocytic anaplasmosis**, symptoms include fever, headache, limb, joint and mucle pain, nausea, vomiting, diarrhea, dry cough, exnthema, multiple organ failure, meningoencephalitis, acute respiratory distress syndrome<br>**Prevalence:** occurs worldwide, 4.000-6000 cases per year in the U.S. (https://www.cdc.gov/anaplasmosis/stats/index.html)	
Ehrlichia	*ewingii*	neutrophils [49]	**Transmission:** ticks (reservoir white-tailed deer)<br>**Disease and symptoms: Human ewingii (granulocytic) ehrlichiosis**, symptoms include high fever, headache, muscle aches, chills, general weakness, fatigue, nausea, vomiting, cough, diarrhea, loss of appetite, confusion, kidney failure, respiratory problems<br>**Prevalence:** limited to the U.S., occurs across the south-central, southeastern, and mid-Atlantic states, infection rates range from 0.3-0.6% [50]	
	chaffeensis	predominantly monocytes, also promyelocytes, neutrophils, lymphocytes [51]	**Transmission:** ticks<br>**Disease and symptoms: Human monocytic ehrlichiosis**, symptoms are comparable to ewingii ehrlichiosis<br>**Prevalence:** 1.500-2.000 cases per year in the U.S., case fatality rate roughly 1% (https://www.cdc.gov/ehrlichiosis/stats/index.html)	
Rickettsia ssp.	TG: *R. typhi*,	ECs, monocytes/MØ	**Transmission:** human body louse (*R. prowazekii*), rodent fleas (*R. typhi*)	

R. prowazekii	, neutrophils [52-54]	**Disease and symptoms: Epidemic typhus** (*R. prowazekii*) and **endemic typhus** (*R. typhi*), high fever, chills, headache, body and muscle aches, rash, cough, nausea, vomiting, pneumonia, liver damage, meningoencephalitis (confusion, unconsciousness, paralysis, neurological symptoms, coma, multi-organ failure; *R. prowazekii* persists, recurrent disease (Brill Zinsser disease)<br>**Prevalence: Epidemic typhus** caused by *R. prowazekii* is re-emerging in foci associated with poor hygiene conditions (e.g., prisons, refugee camps), outbreaks in Cental and South America (Peru), Africa (Burundi, Uganda, Ethiopia, Nigeria, Rwanda), sporadic cases in Northern Africa, Russia, Kazakhstan, and among homeless people in developed countries, sporadic cases of zoonotic origin also occurred in the Eastern US; lethality 20-60% without or wrong antibiotic treatment and <5% with treatment (https://www.ecdc.europa.eu/en/epidemic-louse-borne-typhus/facts)<br>**Endemic typhus** caused by *R. typhi* and occurs worldwide; commonly reported in the Southern US, Southern Europe, Asia, Africa, and Australia, true prevalence unknown due to underdiagnosis or misdiagnosis; lethality 1-5% without or wrong antibiotic treatment (https://www.ecdc.europa.eu/en/epidemic-louse-borne-typhus/facts)	
SFG: > 20 ssp.		**Transmission:** ticks, reservoir: rodents<br>**Disease and symptoms: Spotted fever**, similar to typhus, eschar at the site of entry<br>**Prevalence:** 4.000-6.000 cases per year in the U.S.; 5-10% case fatality for Rocky Mountain Spotted Fever (RMSF; *R. rickettsii*) without antibiotic treatment and roughly 0.5% with treatment (https://www.cdc.gov/rmsf/stats/index.html)	
Orientia ssp.	ECs, DCs, monocytes/MØ [52]	**Transmission:** mite, reservoir: rodents<br>**Disease and symptoms: Scrub typhus**, similar to typhus, eschar at the site of entry<br>**Prevalence:** predominantly occurs in the Asia-Pacific area, 1 million infections estimated worldwide, lethality 30% or ever higher without or with wrong antibiotic treatment [55, 56]	
Coxiella burnetii	EPCs, ECs, fibroblasts, trophoblasts, MØ [57]	**Transmission:** airborne, ticks are discussed as vectors [58]<br>**Disease and symptoms: Q Fever**, symptoms include fever, fatigue, headache, myalgia, loss of appetite, dry cough, thorax pain, shiver, altered consciousness, nausea, vomiting, diarrhea, pneumonia, acute respiratory distress syndrome, liver inflammation, meningitis, endocarditis, pericarditis; in 1-5% of cases, chronic Q-Fever may develop [58]<br>**Prevalence:** occurs worldwide, outbreaks, e.g., in the Netherlands in 2007–2010 [59]	Q-Vax [60] (used only in Australia)

*predominantly extracellular lifestyle; TG: typhus group, SFG: spotted fever group, ECs: endothelial cells, EPCs: epithelial cells, MØ: macrophages, DC: dendritic cells

Supplementary Table S2. Antibiotic resistant bacterial infections in the U.S. reported by the CDC (https://www.cdc.gov/drugresistance/biggest-threats.html).

WHO priority list*	Bacterial pathogen	Resistance	Total cases in the U.S.	Drug resistant cases in the U.S.
1	Acinetobacter ssp. (predominantly *A. baumannii)*	carbapenems, multi-drug resistant	(data not available)	8.500 infections in hospitalized patients and 700 estimated deaths in 2017
	P. aeruginosa	carbapenems, multi-drug resistant	(data not available)	32.600 estimated cases in hospitalized patients and 2.700 deaths in 2017
	Enterobacterales (*E. coli*, *K. pneumoniae*)	carbapenems, multi-drug resistant	(data not available)	13.100 estimated cases in hospitalized patients and 1100 estimated deaths in 2017
	Extended spectrum beta-lactamase (ESBL)-producing Enterobacterales (*E. coli*, *K. pneumoniae*)	carbapenems, multi-drug resistant	(data not available)	197.400 estimated cases in hospitalized patients and 9.100 estimated deaths in 2017
2	Enterococci (*E. faecium*)	vancomycin	(data not available)	54.500 estimated cases in hospitalized patients and 5.400 estimated deaths in 2017
	S. aureus	multi-drug resistant (MRSA)	(data not available)	323.700 estimated cases in hospitalized patients and 10.600 estimated deaths in 2017
	H. pylori	fluoroquinolones, macrolides	(data not available)	(data not available)
	Campylobacter ssp.	fluoroquinolones	(data not available)	448.400 drug-resistant infections and 70 estimated deaths per year
	non-typhoidal *Salmonella*	ciprofloxacin, ceftriaxone, azithromycin	1.35 million infections, 26.500 hospitalizations and 420 deaths per year	212.500 estimated drug-resistant infections and 70 estimated deaths per year
	Salmonella Typhi	ciprofloxacin	5.700 estimated infections and 620 hospitalizations per year (11-21 million infections estimated worldwide)	4.100 estimated drug-resistant infections and <5 deaths per year
	N. gonorrhea	multi-drug resistant; last recommended antibiotic is ceftriaxone	(data not available)	**550.000** estimated drug-resistant infections per year
3	*S. pneumoniae*	one or more antibiotics	> 2 million infections and 150.000 hospitalizations per year	900.000 estimated drug-resistant infections and 3.600 estimated deaths in 2014

	H. influenzae	ampicillin	(data not available)	(data not available)
	Shigella ssp.	ciprofloxacin, azithromycin	450.000 estimated infections per year	77.000 estimated drug-resistant infections and <5 deaths per year
**non-classified**	*M. tuberculosis*	multi-drug resistant	7.860 reported cases in 2021 (estimated 6-8 million cases per year worldwide according to the Global Tuberculosis Report 2021 from the world health organization (WHO))	847 drug-resistant infections and 62 deaths in 2017 (worldwide estimated 0.5 million drug-resistant cases worldwide per year according to the WHO (https://www.who.int/activities/tackling-the-drug-resistant-tb-crisis)
	C. difficile	fluoroquinolones	223.900 infections per year with 12.800 deaths per year	(data not available)
	Streptococcus Group A	erythromycin, clindamycin	1-2.6 million cases of strep throat, 12.500-20.000 invasive infections and 1.250-1.900 deaths per year	5.400 estimated drug-resistant infections and 450 deaths in 2017
	Streptococcus Group B	clindamycin	31.000 severe infections and estimated 1.700 deaths in 2016	13.000 estimated drug-resistant infections and 720 deaths in 2016

* https://www.who.int/news/item/27-02-2017-who-publishes-list-of-bacteria-for-which-new-antibiotics-are-urgently-needed
